# Timing and structure of the Younger Dryas event and its underlying climate dynamics

**DOI:** 10.1073/pnas.2007869117

**Published:** 2020-09-08

**Authors:** Hai Cheng, Haiwei Zhang, Christoph Spötl, Jonathan Baker, Ashish Sinha, Hanying Li, Miguel Bartolomé, Ana Moreno, Gayatri Kathayat, Jingyao Zhao, Xiyu Dong, Youwei Li, Youfeng Ning, Xue Jia, Baoyun Zong, Yassine Ait Brahim, Carlos Pérez-Mejías, Yanjun Cai, Valdir F. Novello, Francisco W. Cruz, Jeffrey P. Severinghaus, Zhisheng An, R. Lawrence Edwards

**Affiliations:** ^a^Institute of Global Environmental Change, Xi’an Jiaotong University, 710054 Xi’an, China;; ^b^State Key Laboratory of Loess and Quaternary Geology, Institute of Earth Environment, Chinese Academy of Sciences, 710061 Xi’an, China;; ^c^Key Laboratory of Karst Dynamics, Ministry of Land and Resources, Institute of Karst Geology, Chinese Academy of Geological Sciences, 541004 Guilin, China;; ^d^Institute of Geology, University of Innsbruck, 6020 Innsbruck, Austria;; ^e^Department of Earth Sciences, California State University, Dominguez Hills, Carson, CA 90747;; ^f^Departamento de Geología, Museo Nacional de Ciencias Naturales, Consejo Superior de Investigaciones Científicas, 28034 Madrid, Spain;; ^g^Instituto Pirenaico de Ecología, Consejo Superior de Investigaciones Científicas, 50059 Zaragoza, Spain;; ^h^Instituto de Geociências, Universidade de São Paulo, 05508-090 São Paulo, Brazil;; ^i^Scripps Institution of Oceanography, University of California San Diego, La Jolla, CA 92093;; ^j^Department of Earth and Environmental Sciences, University of Minnesota, Minneapolis, MN 55455;; ^k^School of Geography, Nanjing Normal University, 210023 Nanjing, China

**Keywords:** Younger Dryas, timing, structure, event phasing, climate dynamics

## Abstract

The Younger Dryas (YD), arguably the most widely studied millennial-scale extreme climate event, was characterized by diverse hydroclimate shifts globally and severe cooling at high northern latitudes that abruptly punctuated the warming trend from the last glacial to the present interglacial. To date, a precise understanding of its trigger, propagation, and termination remains elusive. Here, we present speleothem oxygen-isotope data that, in concert with other proxy records, allow us to quantify the timing of the YD onset and termination at an unprecedented subcentennial temporal precision across the North Atlantic, Asian Monsoon-Westerlies, and South American Monsoon regions. Our analysis suggests that the onsets of YD in the North Atlantic (12,870 ± 30 B.P.) and the Asian Monsoon-Westerlies region are essentially synchronous within a few decades and lead the onset in Antarctica, implying a north-to-south climate signal propagation via both atmospheric (decadal-time scale) and oceanic (centennial-time scale) processes, similar to the Dansgaard–Oeschger events during the last glacial period. In contrast, the YD termination may have started first in Antarctica at ∼11,900 B.P., or perhaps even earlier in the western tropical Pacific, followed by the North Atlantic between ∼11,700 ± 40 and 11,610 ± 40 B.P. These observations suggest that the initial YD termination might have originated in the Southern Hemisphere and/or the tropical Pacific, indicating a Southern Hemisphere/tropics to North Atlantic–Asian Monsoon-Westerlies directionality of climatic recovery.

## Significance

The Younger Dryas (YD) was an ∼1,300-y period of extreme climate that dramatically reversed the course of global warming that brought the last Ice Age to a close. Understanding what mechanisms triggered and terminated this event remains enigmatic, but it is fundamental for gaining insights into the inner workings of Earth’s climate system. In this study, we used a combination of well-dated speleothem and ice-core records to pinpoint the timing of its onsets and terminations in various climatic regimes around the world. We show that the YD event occurred first at high northern latitudes and then propagated southward into the tropical monsoon belt through both atmospheric and oceanic processes, ultimately reaching Antarctica before reversing the course to its eventual termination.

The Earth’s climate system during the last glacial and the deglaciation periods was characterized by a series of millennial-scale extreme events of a global extent (1). The Younger Dryas (YD) (nominally ∼12,900 to 11,600 y before present [B.P.], where present represents 1950 C.E.) was the most recent of these events (2) that has received widespread attention among the scientific community (3). Although some observational and modeling studies have attributed the cause of the YD to variations in the strength of the Atlantic Meridional Overturning Circulation (AMOC) (4, 5), the underlying dynamics regarding its trigger, propagation, and particularly its termination (6) remain poorly understood. This knowledge gap, due in part to lack of high-resolution and precisely dated proxy records of the YD, precludes the precise characterization of its timing, structure, and especially phasing between different climate systems on subcentennial scales.

Recently, key information on the phasing of Dansgaard–Oeschger (DO) events between Greenland and Antarctica has become available via atmospheric methane (CH_4_)-based synchronization of the ice-core oxygen-isotope (δ^18^O) records (7). The new analysis indicates that abrupt onsets of Greenland warming (cooling) lead the corresponding Antarctic cooling (warming) onsets by ∼200 ± 100 y, including during the Bølling warming, implying a north–south propagation of the abrupt climatic signal initiated by changes in the AMOC strength (7) and propagated via a mechanism called the “bipolar seesaw” (8, 9). Subsequent ice-core studies have also revealed that Southern Hemisphere (SH) winds and meridional migrations of the Intertropical Convergence Zone (ITCZ) shifted in phase with the Northern Hemisphere (NH) DO events, suggesting a coupled change of global atmospheric circulation or north to south directionality via the atmosphere (10, 11). These studies focused, however, on DO events of the last glacial period, and much uncertainty remains, therefore, regarding the global teleconnections and dynamics of the YD. Recent developments in U–Th dating have substantially improved the temporal precision of speleothem proxy records (12). The 2σ uncertainty windows of U–Th ages that constrain YD speleothem records are the smallest among all millennial-scale events for samples of similar U content and growth rate, which makes it possible to explore the lead–lag relationships of climate events among different climate systems with unprecedented age control. Speleothem δ^18^O records can, therefore, provide the tightest possible geochronological constraints of any absolutely dated paleoclimate record of the YD and, when combined with the polar ice-core records, constitute an ideal reference archive for characterizing the timing, structure, climatic expression, and signal propagation of the YD event on regional to global scales.

Herein, we report a set of speleothem δ^18^O records of the YD that encompass the North Atlantic, Asian Monsoon (AM), Asian Westerlies (AW), and South American Monsoon domains. We compare these data with a suite of the previously published speleothem and ice-core records to provide a detailed analysis of the YD across different climatic regimes. By focusing on the relative timing and structure of the YD in each region, we aim to identify the dynamic controls associated with its initiation and termination. Within this framework at subcentennial precision, we further discuss the hypothesis of an extraterrestrial-impact trigger for the YD (13). Our results shed light on the underlying dynamics of the YD, particularly with respect to its trigger, transition, and termination.

## Speleothem Samples

In this study, we considerably improved the dating precision and resolution of nine speleothem δ^18^O records from Dongge (D4) (25°17′N, 108°5′E) (14), Shennong (SN29) (28°42′N, 117°15′E) (15), Kulishu (BW-1) (39°41′N, 115°39′E) (16), and Rige (Rige-3) (31°18′N, 97°10′E) (*Materials and Methods*) caves in China; Mawmluh and Cherrapunji (M-1 and Chy-1) (25°16′N, 91°43′) (17) caves in Northeast India; Tonnel’naya Cave (TON-1) (38°24′N, 67°14′E) (18) in Uzbekistan; Jaraguá Cave (JAR-7) (21°05′S, 56°35′W) (19) in Brazil; and Seso Cave (SE09-6) (42°27′N, 0°02′E) (20) in Spain (*SI Appendix*, Fig. S1). The climatic significance of these records has been well explained in the aforementioned references. In short, the speleothem δ^18^O records from monsoon domains reflect the convective intensity of monsoon circulation (21, 22), the Seso δ^18^O record from Spain is a temperature proxy similar to the Greenland ice-core δ^18^O record (20), and the Tonnel’naya δ^18^O record from Uzbekistan depicts changes in large-scale atmospheric circulation in the AW domain (18).

The speleothem chronologies reported here are based on extensive U–Th dating (192 dates) by a recently improved technique (12). Subsamples for dating were obtained by drilling the polished stalagmite section along the growth axis with a carbide dental burr. The dating work was performed at the Isotope Laboratory of Xi’an Jiaotong University, using multicollector inductively coupled plasma mass spectrometry (MC-ICP-MS) (*Materials and Methods*). Typical age uncertainties (2σ) vary between 15 and 40 y for most key intervals (*SI Appendix*, Table S1). The age models of speleothem δ^18^O records were constructed using OxCal (23) and StalAge software (24), as well as the incorporation of annual band counting when resolvable through confocal microscopy (*SI Appendix*, Figs. S2 and S3). A total of ∼5,100 oxygen-isotope (δ^18^O) subsamples were analyzed at the University of Innsbruck, Austria (sample SE09-6), and Xi'an Jiaotong University, China (the rest of samples) (*SI Appendix*, Table S2). The δ^18^O values are reported in per mil (parts per thousand, ‰) deviations, relative to the Vienna Pee Dee Belemnite (VPDB) standard. The long-term reproducibility for δ^18^O measurements over the course of this study on both laboratories was typically ∼0.1‰ (1σ).

## Results and Discussion

### Speleothem Record from the North Atlantic Region.

**Fig. 1.**
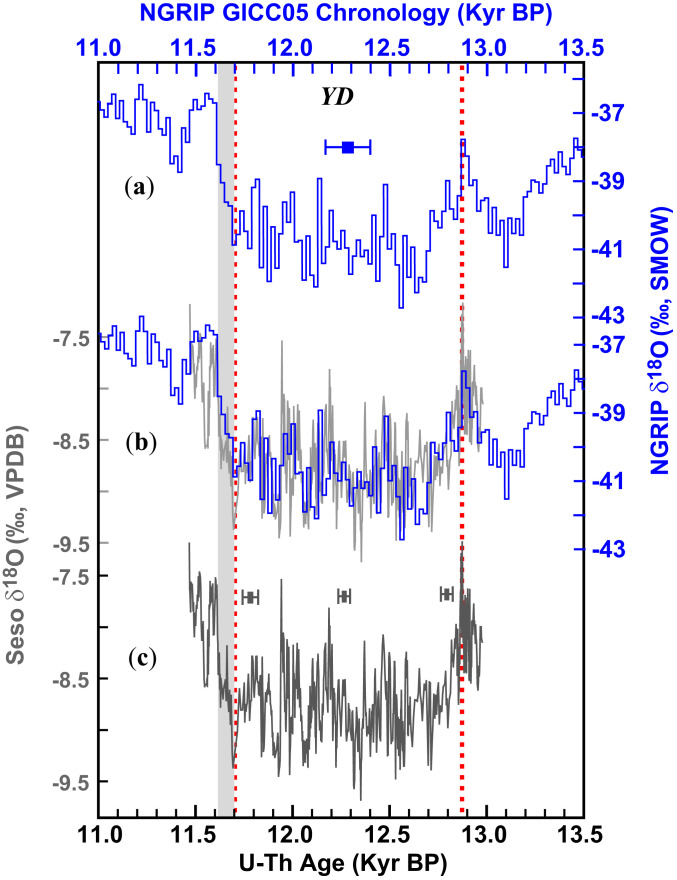
Comparison between Greenland NGRIP ice-core and Seso speleothem δ^18^O records. (*A*, *C*) Greenland NGRIP (25) and Seso speleothem δ^18^O records (this study), respectively. (*B*) Comparison between NGRIP (blue) and Seso (gray) δ^18^O records. The error bars show the typical age error of each YD record (color-coded). The two vertical red dashed lines depict the initial onset (the abrupt drop at ∼12,870 ± 30 B.P.) and initial termination (initiated at ∼11,700 ± 40 B.P.) of the YD based on correlation of distinct features at subcentennial precision. The gray bar shows the YD termination excursion from ∼11,700 to ∼11,610 B.P. Kyr BP, 1 × 10^3^ B.P.

The Greenland ice-core chronology (GICC05) uncertainty of the North Greenland Ice Core Project (NGRIP) is about ±100 to 140 (2σ) yr for the YD interval (25), which precludes any direct correlations with other climate records at subcentennial precision. We therefore used the Seso Cave δ^18^O record from Spain, which has been shown to be a robust temperature proxy for the YD interval and is strongly correlated to the Greenland δ^18^O record (20), to provide key chronological constraints on the YD in the circum-North Atlantic region. We improved the Seso (SE09-6) δ^18^O record substantially in both dating precision (±20 to 40 y; 2σ) and temporal resolution (∼2 y) (Fig. 1 and *SI Appendix*, Fig. S2 and Tables S1 and S2). This Seso δ^18^O record confirms a robust correlation with NGRIP within ±20 to 40 y, suggesting that the NGRIP chronology (on the GICC05 time scale) around the YD is more precise than the quoted absolute error of ±100 to 140 y (Fig. 1). This verification allows for the correlation/synchronization of global YD records to the North Atlantic region at an unprecedented precision of ±20 to 40 y, which is approximately a threefold improvement over the ±100 to 140 y uncertainty window in GICC05. The NGRIP and Seso Cave records show an impressive match in their δ^18^O pattern, down to centennial-scale variations (Fig. 1). Of note are two anchor points in both records: 1) the first large and abrupt drop in δ^18^O (∼2‰ within ∼20 y) at 12,870 ± 30 B.P. as a clear sign of the YD onset; and 2) an ∼90-y, two-step δ^18^O excursion from ∼11,700 to ∼11,610 B.P. as a marker of the YD termination/Holocene onset (Fig. 1), beginning at 11,700 ± 40 B.P. These quoted uncertainties are based on the age model of the Seso record (±30 to 40 y) (*SI Appendix*, Fig. S2). The timings of the two anchor points are consistent with corresponding “breakpoints” determined by fitting a ramp trend ("RAMPFIT") (26) and a trend change ("BREAKFIT") (27) models to data (*SI Appendix*, Fig. S4 and Tables S3–S5). Additionally, the timing further agrees well, within decadal uncertainty (±30 to 40 y), with a hydrogen-isotope record of lipid biomarkers from varved lacustrine sediments of Meerfelder Maar, Germany (28), as well as the European tree-ring chronology in terms of the timing of the abrupt Holocene onset (29). In the following discussion, the two anchor points at the onset and termination of the YD provide a basis for global YD correlation/synchronization at subcentennial precision.

### Speleothem Records from the AM-AW Region.

The AM is a vast climate system, which transports large amounts of moisture and heat northward from northern Australia and the Mascarene High across the Indian Ocean into India, southeastern China, and as far as northeastern China and Japan (30). The AW is another large climate system extending from the eastern Mediterranean to western China, which is dynamically coupled with the AM on a wide range of time scales (18, 31). A strong link is also recognized between North Atlantic and AM-AW millennial events, including the YD (18, 22, 32). Nevertheless, high-resolution and precisely dated YD records (with subcentennial precision) are rare in AM-AW domains. The correlation/synchronization of proxy records from distant climatic regimes is generally based on matching the “midpoints” of corresponding climate shifts (7, 32)—a strategy that is generally suitable when climate records are of low resolution with less precise age constraints. This approach assumes temporal synchronicity, however, which is inconsistent with emerging evidence. Particularly, a growing number of high-resolution speleothem records from the AM region exhibit a more gradual onset and termination of millennial-scale events, including the YD. In this regard, they are to some extent more similar to the gradual shifts of the Antarctic, rather than the abrupt changes characteristic of Greenland climate (33–35). Results of midpoint matching further imply that the initial shifts in the low-latitude AM regions led the hydroclimate change in Greenland, suggesting that the trigger plausibly resides in the low latitudes than the North Atlantic domain (36) (*SI Appendix*, Fig. S5). The apparent phase relationship thus contradicts the prevailing notion of a climate dynamic trigger in the North Atlantic (37, 38). Alternatively, one can employ a synchronization strategy that utilizes breakpoints rather than midpoints (36), in line with the assumption of synchroneity between the abrupt Greenland hydroclimate change and the initial AM-AW response. Whether the breakpoint or midpoint strategy is valid to the case of the YD has remained an open question until now, due to age-model limitations. Similarly, a detailed study of Greenland Stadial 20 and Greenland Interstadial (GIS) 20 (∼73,000 B.P.) could not provide a direct test, due to comparably large absolute age uncertainties at that time (>200 y for speleothem records and >1,000 y for Greenland ice-core records) (36). Our updated YD chronologies are thus critically important, as they allow statistically robust validation of correlation strategies.

**Fig. 2.**
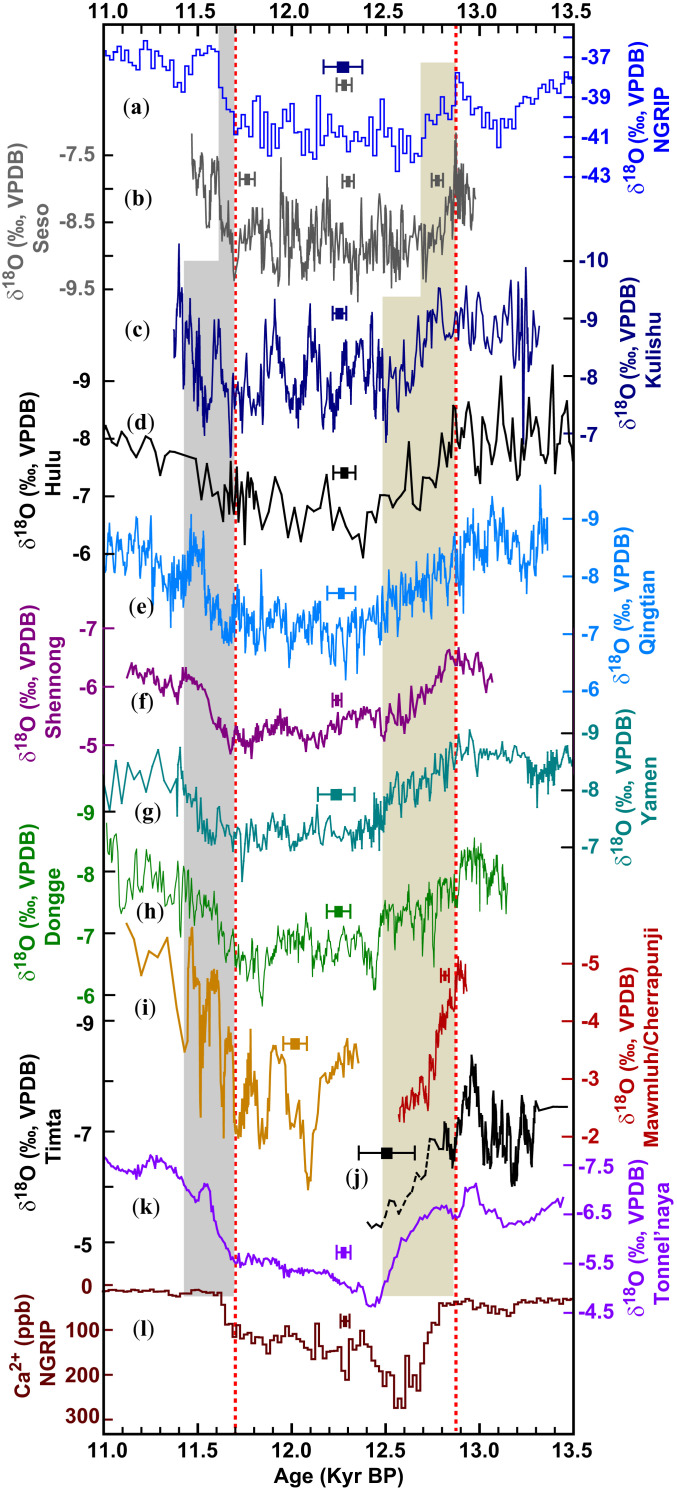
Comparison of δ^18^O records from the North Atlantic, East AM, Indian Monsoon, and AW domains. (*A*, *B*) NGRIP δ^18^O on GICC05 chronology (25) and Seso δ^18^O record from the North Atlantic, respectively. (*C*–*H*) Kulishu, Hulu (38), Qingtian (39), Shennong, Yamen (40), and Dongge δ^18^O records from the East AM domain, respectively. (*I*) Mawmluh (yellow) and Cherrapunji (marron) δ^18^O records from the Indian Monsoon domain. (*J*) Timta δ^18^O record from the Indian Monsoon domain (41). (*K*) Tonnel’naya δ^18^O record from the AW domain. (*L*) NGRIP Ca^2+^ on the GICC05 chronology (1) (*SI Appendix*, Fig. S1). Speleothem records are from this study except for those indicated by references. Error bars depict the typical age error of each record. Two vertical red dashed lines depict the initial onset (12,870 ± 30 B.P.) and termination (11,700 ± 40 B.P.) of the YD based on Seso and NGRIP δ^18^O records. The vertical bars show durations of full-onset (beige) and termination (gray) excursions of the YD. Kyr BP, 1 × 10^3^ B.P.

The speleothem δ^18^O records reported here were obtained from the AM domain, including the East AM subsystem (Dongge, Shennong, and Kulishu caves in China) the Indian Monsoon subsystem (Mawmluh and Cherrapunji caves in Northeast India), and AW domain (Tonnel’naya Cave in Uzbekistan). Together with the previously published Hulu (38), Qingtian (39), Yamen (40), and Timta (41) records, these datasets allow us to directly compare the YD in the mid- to low-latitude AM-AW domain to the North Atlantic realm at subcentennial precision. On the basis of distinctive stable-isotope features of well-dated records, the initial onset of the YD in the AM-AW domain is constrained by the Cherrapunji δ^18^O records at ∼12,890 ± 20 B.P. (*SI Appendix*, Figs. S3 and S4). The timing of this initial onset is coherent with the North Atlantic counterparts within a few decades. Additionally, it corroborates the hypothesis of a YD trigger residing in the North Atlantic and fast atmospheric propagation (on decadal scale) of the signal to the AM-AW climate system (10, 11). In contrast, the full YD onset excursion lasts longer in the AM-AW records (∼350 y) than in corresponding North Atlantic speleothems (<200 y) (Fig. 2 and *SI Appendix*, Fig. S4), implying a northern high-latitude to low-latitude directionality of the abrupt climatic signal and hinting toward oceanic reorganizations as an intermediate dynamic (given that atmospheric propagation would be much faster), including changes in the land–sea temperature gradient (22, 30) and/or meridional tropical sea–surface temperature (SST) gradients (42, 43), as well as possible impacts from SH climate changes via the “bipolar seesaw” and concomitant feedbacks (7–9, 11, 33, 34).

The start of the YD termination in the AM-AW records is constrained by the δ^18^O maxima of the breakpoints in well-dated records: ∼11,670 ± 50 B.P. in the Shennong record, ∼11,710 ± 40 B.P. in the Tonnel’naya record, and ∼11,680 ± 90 B.P. in the Kulishu record (Fig. 2 and *SI Appendix*, Figs. S2 and S4 and Tables S3–S5). These ages are consistent with the resumption of speleothem growth around 11,675 ± 65 B.P. at Kinderlinskaya Cave in the Ural Mountains, which reflects the midlatitude degradation of permafrost associated with the YD termination (44). While the initial termination of the AM-AW YD is effectively synchronous with the North Atlantic at ∼11,700 B.P., the full AM-AW termination excursion (∼300 y) lasted considerably longer there than in the North Atlantic realm (∼90 y) (Fig. 2 and *SI Appendix*, Fig. S4), suggesting a dynamic relation akin to the YD onset: a northern high-latitude to mid- to low-latitude directionality (45) via both atmospheric (e.g., northward shifts of ITCZ and midlatitude westerly winds in both hemispheres and changes in the tropical Hadley circulation) (10, 46, 47) and oceanic processes (e.g., changes in AMOC, subsequent South Ocean temperature and the feedbacks via the bipolar seesaw) (8). In addition, the results also provide a direct test for correlation strategies and support the matching of breakpoints over midpoints. By contrast, the conventional midpoint matching would require a shift of the North Atlantic records toward younger ages by more than 100 y, which is beyond the 2σ error margin.

### Speleothem Records from the Tropical Pacific and SH.

**Fig. 3.**
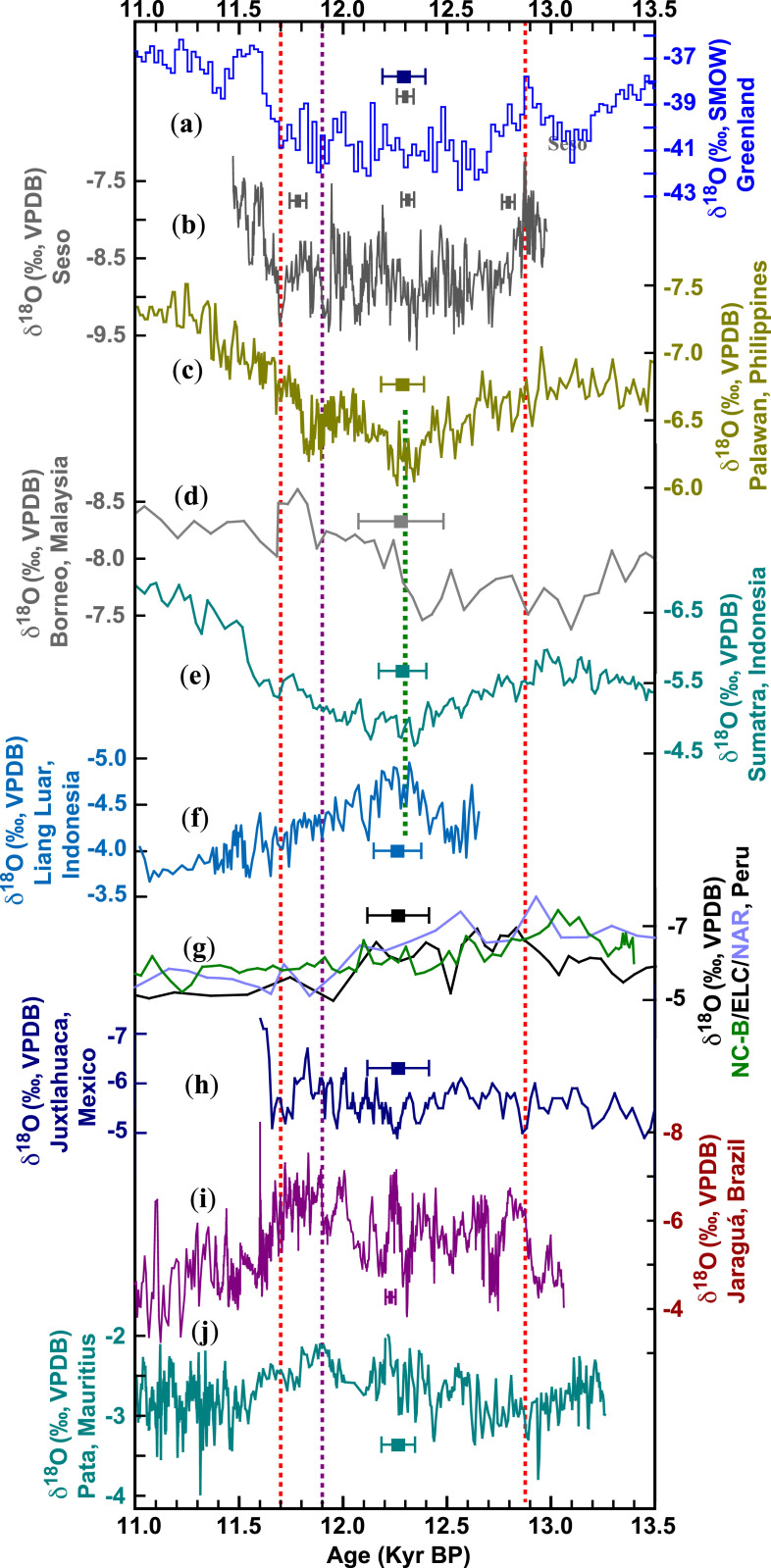
Comparison of speleothem δ^18^O records from North Atlantic and tropical Pacific regions. (*A*, *B*) NGRIP δ^18^O on the GICC05 chronology (25) and Seso δ^18^O record from the North Atlantic region, respectively. (*C*–*F*) Palawan (48), Borneo (49), Sumatra (50), and Liang Luar (51) records from the western tropical Pacific and nearby regions. (*G*, *H*) are NC-B/ELC-B/NAR-C (53, 54) and Juxtlahuaca (52) records near the eastern tropical Pacific. (*I*) Jaraguá record from the South American Monsoon domain (this study). (*J*) Patate record from the South Indian Ocean (59). Error bars depict the typical age error of each record. Two vertical dashed red lines mark the initial onset (12,870 ± 30 B.P.) and initial termination (11,700 ± 40 B.P.) of the YD. The vertical purple line indicates the initial termination in two SH records, (*I*) and (*J*), at ∼11,900 B.P., consistent with the WAIS record (Fig. 4). The vertical dashed green line indicates the beginning of the YD termination excursion in the western tropical Pacific at ∼12,300 B.P. Kyr BP, 1 × 10^3^ B.P.

Several speleothem records spanning the YD are available from the tropical Pacific. These include records from Palawan (48), Borneo (49), Sumatra (50), and Liang Luar (51) from the western tropical Pacific and Juxtlahuaca (52), Cueva del Diamante (ELC-B)/Cueva del Diamante (NAR-C) (53), and Cueva del Tigre Perdido (NC-B) (54) from near the eastern tropical Pacific. The western tropical Pacific δ^18^O records are generally interpreted as a hydroclimatic proxy indicating rainfall amount (48–51). A common feature of the YD in these tropical records is their gradual change, relative to those from the North Atlantic (Fig. 3). Particularly, the gradual termination excursion toward the end of the YD appears to commence at ∼12,300 B.P., significantly earlier than its counterparts in the North Atlantic and AM-AW regions (Figs. 2 and 3 and *SI Appendix*, Fig. S4), suggesting a long-term continuous increase in rainfall or convection (Palawan, Borneo, and Sumatra sites) and a shift in Australian–Indonesian summer monsoon intensity at the Liang Luar Cave site in SH. SST also appears to increase in the region since the mid-YD (55, 56). On the other hand, the Juxtlahuaca and ELC-B/NAR-C/NC-B δ^18^O records near the eastern tropical Pacific are also interpreted to indicate rainfall amount (52–54), but their YD termination excursions are distinctive compared with the western tropical Pacific. The YD variation in the Juxtlahuaca record is small without a clear trend, and the ELC-B/NAR-C/NC-B records all suggest a slightly decreasing trend in rainfall toward the end of the YD at ∼11,610 B.P. (Fig. 3). The east–west tropical precipitation gradients, similar to SSTs, reflect the El Niño Southern Oscillation (ENSO) variability. This east–west pattern across the tropical Pacific and adjacent regions may be conceptually viewed as a trend toward a more La Niña-like state (57) with increased rainfall (convection) or temperature (58) in the west and stable/decreased rainfall in the east. Intriguingly, this trend was established already by ∼12,300 B.P., long before the initiation of the YD termination in the North Atlantic and AM-AW domains at ∼11,700 B.P.

Two well-dated YD records from the SH—the Jaraguá record from the South American Monsoon domain (this study) and the Patate record from Southern Indian Ocean (59) (*SI Appendix*, Fig. S1)—are similarly characterized by gradual shifts (Fig. 3). Also of note is that their earlier termination started at ∼11,950 to 11,850 B.P. (Fig. 3 and *SI Appendix*, Fig. S4). The termination excursion in the Jaraguá δ^18^O record manifests from a progressive weakening of South American Monsoon intensity or decreasing rainfall (19), and the Patate δ^18^O values decrease during the termination excursion is linked to the ITCZ intensification and resultant stronger convective activity (59).

### YD Phasing Relations between Antarctica and Greenland.

Comparisons between Antarctic and Greenland ice-core records demonstrated that abrupt Greenland warmings (coolings) led the corresponding onset of Antarctic coolings (warmings) by ∼200 ± 100 y (2σ) for DO events (9, 11). In this study, we have confirmed the NGRIP ice-core chronology (GICC05) to within ±20 to 40 y across the YD (Fig. 1). The high-accumulation West Antarctic Ice Sheet (WAIS) Divide Ice Core (WDC) provides high-resolution atmospheric CH_4_ and ice δ^18^O data (a first-order temperature/circulation proxy) at relatively high age precision (±80 to 110 y for the YD) (60, 61), providing an opportunity to test directly the hypothesis of interpolar phasing of the YD based on absolute age constraints.

**Fig. 4.**
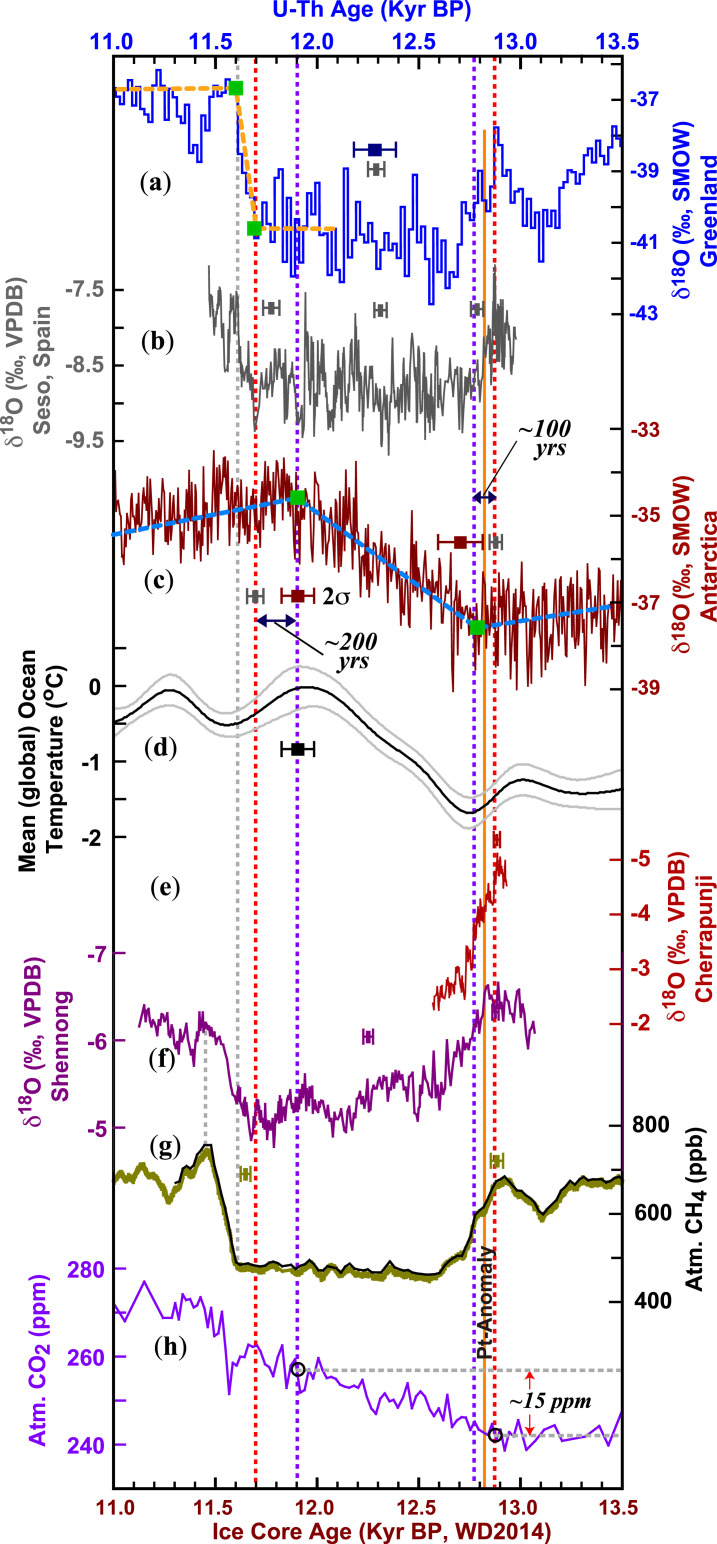
Interpolar phasing. (*A*) NGRIP δ^18^O on the GICC05 chronology (25). Orange dashed lines and green squares depict trends and breakpoints of the record. (*B*) Seso δ^18^O record (this study). (*C*) Antarctic WDC δ^18^O record on the WD2014 chronology (61). Blue lines and green squares depict trends and breakpoints of the record, respectively. (*D*) Mean global ocean temperature record (62). The gray lines indicate uncertainty. (*E*) East AM δ^18^O record (Shennong record; this study). (*F*) Indian Monsoon δ^18^O record (Cherrapunji record; this study). (*G*) Atmospheric CH_4_ records from the WDC ice core (olive, ref. 60; black, ref. 61). (*H*) Atmospheric CO_2_ record from the WDC ice core (on WD2014 chronology) (84). Error bars depict the typical age error of each record, except for CH_4_ records, which show the uncertainty of the ice–gas age difference (61). The blue and gray errors for the NGRIP δ^18^O record depict NGRIP age error (GICC05 chronology) and the error based on synchronization to the Seso chronology, respectively. Two vertical red dashed lines depict the initial onset (12,870 ± 30 B.P.) and initial termination (11,700 ± 40 B.P.) of the YD in the NGRIP and Seso records. Two vertical purple lines indicate two breakpoints in the WDC δ^18^O record at ∼11,900 and ∼12,770 B.P. Two vertical gray dashed lines indicate the abrupt jump in North Atlantic temperature at ∼11,610 B.P. and the peak of AM and CH_4_ around ∼11,450 B.P. at the end of the YD. The solid orange line depicts the Pt-anomaly in the GISP2 ice core (73) at ∼12,820 B.P. on GICC05 chronology (1). Two horizontal dashed gray lines in (*H*) depict an ∼15-ppm increase (red arrows) of atmospheric CO_2_ since the initial onset of the YD. Kyr BP, 1 × 10^3^ B.P.

We identified two breakpoints (*Materials and Methods*) in the WDC δ^18^O record on the WD2014 chronology (61) during the YD interval: at ∼12,770 ± 110 B.P. and ∼11,900 ± 80 B.P. (Fig. 4 and *SI Appendix*, Fig. S4). These boundaries are well supported by the mean global ocean temperature reconstruction, which is presumably synchronous with Antarctic δ^18^O changes (Fig. 4) because the parameter is biased toward SH surface temperature due to the larger ocean volume and areal extent (62). The breakpoint at ∼12,770 ± 110 B.P. marks the initial onset of the YD in Antarctica, which is ∼100 y after the initial onset of the YD in Greenland at ∼12,870 ± 30 B.P. and ∼120 y after the initial drop in atmospheric CH_4_ at ∼12,890 ± 30 B.P. (the error of ice/gas age difference) (51) determined from the same ice core. This temporal relation is similar to the interpolar phasing observed previously for DO events (9, 11). In contrast, the phasing relation of the YD termination between these regions appears to be the opposite. The breakpoint at ∼11,900 ± 80 B.P. in the WDC δ^18^O record occurs ∼200 ± 120 y before the initial termination of the Greenland YD at ∼11,700 ± 40 B.P. (Fig. 4). Other high-resolution Antarctic ice-core records are broadly consistent with the WDC record, except Dome Fuji, which does not show such a breakpoint around the YD termination (*SI Appendix*, Fig. S6). The above phasing relation can be further tested by the WDC CH_4_ records on the same chronology (WD2014), due to a small uncertainty (±30 y) in the WDC ice–gas age difference (61) and a strong correlation between CH_4_ and AM/Greenland δ^18^O records (9, 63–65) controlled by the extent of wetlands and thus CH_4_ emissions (66). During the termination excursion, the breakpoint in the CH_4_ record is around 11,610 B.P., about 100 y later (rather than earlier) than the initial termination in AM and North Atlantic records. A closer look at these records reveals that the CH_4_ values were virtually invariant (∼500 parts per billion) during the YD between ∼12,620 and 11,610 B.P., while the AM and North Atlantic climate exhibited considerable centennial-scale oscillations (*SI Appendix*, Fig. S7). This apparent decoupling may explain the delayed CH_4_ termination rise from ∼11,610 to ∼11,480 B.P., which is closely coupled to the AM intensification significantly above the threshold of the mean YD value (*SI Appendix*, Fig. S7). Additionally, the gradual centennial-scale AM intensification from ∼11,610 to ∼11,450 B.P. contrasts with the rather abrupt decadal-scale North Atlantic temperature jump at ∼11,610 B.P., suggesting an atmospheric role (10, 11, 46, 47) on decadal scales via coupled global atmospheric circulation, aforementioned SH changes (33, 34), and oceanic controls (7, 11, 42, 43) on centennial scales in driving the AM (and CH_4_) termination in response to the abrupt change in the northern high latitudes.

An extraterrestrial impact has also been hypothesized as a plausible trigger for the YD-onset and hydroclimatic anomaly (13). A large array of proxy data from the YD boundary (YDB) layer from various climate systems supports this “YD Impact Hypothesis” (67, 68), and a modeled YDB age of this extraterrestrial event based on a large set of ^14^C dates suggests a time range of the YDB between 12,835 and 12,735 B.P. (69), as calibrated by IntCal13 (70), or between ∼12,875 and 12,775 B.P., as calibrated by IntCal20 (71). While this YDB age range agrees within error margin with the YD onset at 12,870 ± 30 B.P., a most recent simulation work demonstrates that this set of ^14^C samples are extremely unlikely to have been deposited synchronously, calling into question the YD Impact Hypothesis (72). As such, it would be ideal to find the presumably large-scale extraterrestrial signal directly from Greenland ice cores to test its causal link to the YD event without the restraint of age uncertainty. Indeed, an ∼20-y-long Pt-anomaly was identified in the Greenland Ice Sheet Project (GISP2) ice core (73), which was attributed to injections of Pt-rich dust from the event and subsequent deposition at a depth of 1,712.375 to 1,712.000 m, or at ∼12,820 B.P., based on synchronization to the GICC05 chronology (1) (Fig. 4 and *SI Appendix*, Fig. S8). A closer look, however, found that the immediate hydroclimatic impact, if any, was likely minor as inferred from GISP2 δ^18^O record (corresponding to a <1‰ drop; *SI Appendix*, Fig. S8). In the same ice core, the Pt-anomaly occurred at the middle of a gradual increase in Ca^2+^ (dust proxy) from ∼1,714.00 to 1,709.90 m (∼12,870 to 12,765 B.P. on GICC05 chronology) without disrupting the course (*SI Appendix*, Fig. S8). Provided that the GISP2 and NGRIP records were synchronized precisely (1), the Pt-anomaly did not disrupt NGRIP and AM δ^18^O records either (*SI Appendix*, Fig. S8). Additionally, there is no clear evidence that the YD-onset excursion has been interrupted substantially around the time of the Pt-anomaly, either in the South American Monsoon or in tropical records (Figs. 2–4 and *SI Appendix*, Fig. S3). These observations are thus inconsistent with the hypothesis that the extraterrestrial event triggered the YD unless the extraterrestrial event did not leave any imprints in the Greenland ice core, which would be also inconceivable. Moreover, the YD as a millennial-scale perturbation during the last deglaciation has a previous analog: a YD-like event occurred at ∼245,000 B.P. during glacial termination-III (the third to the last deglaciation) (64, 74). Based on this paleoanalog and the preponderance of geochronological data, we contend that the YD Impact Hypothesis remains untenable and offers a less parsimonious explanation for the global timing and structure of the YD event, and the data presented here provide a precise timing framework for further research in the area.

### The Trigger of the YD and Climate Dynamics.

The initial onset of the YD, inferred from the abrupt North Atlantic change at ∼12,870 ± 30 B.P., is synchronous within the error of changes in the AM-AW domain and likely the South American Monsoon and tropical hydroclimate regions as well (Figs. 2 and 3). We have argued that the phasing provides direct evidence that mid- to low-latitude hydroclimatic changes occurred within a few decades of the abrupt change in the North Atlantic, suggesting a fast propagation via meridional migration of ITCZ, Hadley circulation, and westerly jet stream (10, 11, 46, 47). Conversely, the fact that AM-AW and tropical hydroclimates show a considerably longer YD-onset excursion than the North Atlantic realm suggests a northern high-latitude to mid- to low-latitude directionality of the YD onset, which highlights the role of oceanic processes (e.g., changes in Southern Ocean temperature) in responding to the abrupt change in the AMOC strength via the bipolar seesaw (7, 8, 11, 37) and their subsequent impacts on the AM-AW (33, 34, 42, 43). This mechanism is consistent with the notion that monsoon climate is fundamentally driven by changes in land–sea thermal gradients; hence, oceanic dynamics and especially SST changes are critical controls on monsoon variability. Moreover, the initial YD onset in mid to low latitudes leads the corresponding Antarctic warming onset by ∼100 y. The relative timing and directionality of YD climate expressions indicate a global signal propagation from northern high latitudes to mid- to low-latitude systems to the southern high latitudes, for which the magnitude of temporal offsets and geographic reach can only be explained through a combination of atmospheric and oceanic processes (7, 10, 11).

The termination excursion of the YD is rather distinctive in contrast to the DO events (7, 11). The YD termination in the AM-AW domain begins at ∼11,700 B.P., synchronous within uncertainty with the abrupt northern high-latitude hrydroclimate change in North Atlantic records. The more gradual change observed in the AM-AW records suggests a northern high-latitude to mid- to low-latitude directionality, similar to the onset of the event. However, the gradual termination of the YD in the western tropical Pacific seems already to have initiated by ∼12,300 B.P. (Fig. 3) and is characterized by a subsequent shift toward a more La Niña-like state. The relatively early onset of hydroclimatic shifts in the ENSO domain may indicate that the trigger of the YD termination originated in the tropics (57). The switch from an El Niño-like to a La Niña-like state has long been recognized across the end of the YD from marine records of the tropical Pacific (57, 58). The key observation from speleothems lies in the timing of the YD initial termination in the tropical Pacific prior to the North Atlantic. The western tropical Pacific is a major source of heat and moisture for extratropical regions (75, 76). A shift toward a more La Niña-like state might have ultimately induced a persistently positive North Atlantic Oscillation (NAO)/Atlantic Multidecadal Oscillation (AMO) state (77), thus strengthening the AMOC (78, 79). Nevertheless, the causal sequence or phasing of climate events between tropics and the North Atlantic remains a challenging issue for modeling approaches (80–82), and the observational constraints presented here supported by their excellent chronology to understand timing and phasing are hence valuable for future model simulations.

The initial termination of the YD also appears to be early in the SH: at ∼11,950 ± 30 B.P. in the Jaraguá record from the South American Monsoon domain and at ∼11,850 ± 100 B.P. in the Patate record from the South Indian Ocean (Fig. 3). The high-resolution WDC record provides an Antarctic δ^18^O record on a well-constrained chronology, which shows an initial YD termination at 11,900 ± 80 B.P. based on WD2014 chronology or ∼11,900 ± 30 based on the CH_4_-AM synchronization and the small uncertainty (±30 y) of the WDC ice–gas age difference (Fig. 4 and *SI Appendix*, *Text* and Fig. S7). A close inspection of the AM records shows that after ∼11,900 B.P., the AM exhibits a weak increasing trend (*SI Appendix*, Fig. S9), which might be a response to the SH change (33, 35). Theoretically, SH and/or Antarctic changes may result in a warmer NH and particularly North Atlantic through interhemispheric heat redistribution (83). Another mechanism involves the direct radiative forcing (84), particularly an atmospheric CO_2_ increase of ∼15 parts per million (ppm) from the initial YD onset at ∼12,870 B.P. (∼242 ppm) to ∼11,900 B.P. (∼257 ppm) (84) (Fig. 4), which reached the threshold of a 15-ppm increase in atmospheric CO_2_ (85). Mechanistically, an ∼15-ppm CO_2_ rise during a millennial-scale event is sufficient to alter the atmospheric moisture transport across Central America and, in turn, modulate the North Atlantic freshwater budget, ultimately resulting in a transition from a weak to a strong AMOC mode (85).

Based on the aforementioned time series, hydroclimate changes observed in the western tropical Pacific and/or SH might have initiated the YD termination via a La Niña-like state and/or cooling in SH. These events plausibly acted as precursors to a gradual shift (86) in the AMOC system, which ultimately reached a tipping point (87), allowing for the resumption of a strong AMOC mode (5) that led to the abrupt temperature rise in the North Atlantic realm. As such, the underlying climate dynamics during the YD termination manifested through an SH and/or tropics to northern high-latitude directionality. The spatiotemporal constraints herein thus provide an interpretive framework for future studies to explore the dynamics of global ocean–atmosphere teleconnections between these systems, associated with the unique signal propagations of the YD event.

## Conclusions

The YD datasets presented herein are characterized by subcentennial age precision, allowing for a robust analysis of the timing and structure of the YD event. Our results provide insights into the dynamics and succession of climate change across the most recent and comprehensively studied stadial of the Pleistocene. The initial YD onset occurred at ∼12,870 ± 30 B.P. in the North Atlantic, synchronous with the AM-AW domain within decadal uncertainty, implying a fast atmospheric propagation. A possible extraterrestrial impact event at ∼12,820 B.P. inferred by Pt-anomaly in the GISP2 ice core appears to lag the initial onset of the YD by ∼50 y without apparent disruption on the hydroclimate trend, suggesting that this event might not be the trigger for the YD onset. In contrast, the longer and more gradual YD-onset excursion in the AM-AW domain suggests an oceanic reorganization in response to the abrupt North Atlantic climate change. The initial Antarctic shift appears to lag the initial YD onset in the North Atlantic and the AM-AW by ∼100 y. Collectively, these observations demonstrate a northern high-latitude to mid- to low-latitude to southern high-latitude directionality during the onset of the YD via both atmospheric and oceanic processes. The initial termination of the YD occurred at ∼11,900 ± 80 B.P., as inferred from the Antarctic δ^18^O record, or possibly earlier (∼12,300 B.P.) if indicated by the trend toward a more La Niña-like state in the western tropical Pacific, as well as a first weak increase in AM intensity. The abrupt termination of the YD in the North Atlantic realm occurred from ∼11,700 to 11,610 B.P. and in the AM-AW from ∼11,700 to 11,450 B.P. Although the dynamic relationship between the North Atlantic and the AM-AW appears similar to the YD-onset excursion, the initial trigger might reside in either the tropics, the SH, or both, suggesting a tropical-SH to North Atlantic–AM-AW directionality. These spatiotemporal constraints thus provide an interpretive framework for future empirical and modeling studies to pinpoint the underlying mechanism(s), which presumably are different from earlier DO events.

## Materials and Methods

### Paleoclimate Records.

Nine speleothem samples were selected for this study. The U–Th dating precision and temporal resolution of oxygen-isotope data (δ^18^O) were considerably improved. The sample information and cave settings are as follows: D4 from Dongge Cave, China (25°17′N, 108°5′E) (14); SN29 from Shennong Cave, China (28°42′N, 117°15′E) (15); BW-1 from Kulishu Cave, China (39°41′N, 115°39′E) (16); Rige-3 from Rige Cave, China (32°13′N, 97°12′E); M-1 from Mawmluh Cave, India; Chy-1 from Cherrapunji Cave, India (25°16′N, 91°43′E) (17); TON-1 from Tonnel’naya Cave, Uzbekistan (TON-1) (38°24′N, 67°14′E) (18); JAR-7 from Jaraguá Cave, Brazil (21°05′S, 56°35′W) (19); and SE09-6 from Seso Cave, Spain (42°27′N, 0°02′E) (20) (*SI Appendix*, Fig. S1). Rige Cave is located near Yushu City in the east-central Tibetan Plateau (4,252 m above sea level) (*SI Appendix*, Fig. S1). The mean annual precipitation in the area is ∼460 mm, of which ∼85% falls during summer (June to September), when the Indian summer monsoon prevails. The mean annual temperature measured at Yushu meteorological station, 85 km north of the cave at 3,682 m above sea level, is ∼4 °C. The cave temperature was ∼2 °C when we collected the sample Rige-3 in June 2019. The sample Rige-3 is ∼19 cm long with a diameter of ∼8 cm. The YD onset is at the depth of ∼152 to 155 mm from the top. Existing datasets used in this study (*SI Appendix*, Fig. S1) include 1) speleothem δ^18^O records from Hulu (38), Qingtian (39), and Yamen (40) caves from the East AM domain; Timta Cave from Indian Monsoon domain (41); Palawan (48), Borneo (49), Sumatra (50), and Liang Luar (51) caves from the western tropical Pacific; ELC-B/NAR-C (53), NC-B (54), and Juxtlahuaca (52) caves near the eastern tropical Pacific; Patate Cave from the South Indian Ocean (PATA-1) (59); and 2) ice-core records from Greenland sites NGRIP and GISP2 (25), Antarctic sites WDC (61, 62), Siple Dome (88), EPICA Dome C (EDC), Talos Dome, EPICA Dronning Maud Land (EDML), and Dome Fuji (9, 88).

### U–Th Dating Method.

Stalagmites were halved along the growth axis and polished. About 20 to 150 mg of powder was drilled near the central axis for each U–Th subsample. These subsamples were obtained by drilling the polished stalagmite section along the growth axis with a carbide dental burr. U–Th dating work was performed at the Isotope Laboratory, Xi’an Jiaotong University, using MC-ICP-MS (Neptune-plus; Thermo-Finnigan). We used standard chemistry procedures to separate U and Th for dating (89). A triple-spike (^229^Th–^233^U–^236^U) isotope-dilution method was employed to correct for instrumental fractionation and determine U–Th isotopic ratios and concentrations. The instrumentation, standardization, and half-lives are reported in refs. 12, and 90. All U–Th isotopes were measured on a MasCom multiplier behind the retarding potential quadrupole in peak-jumping mode. We followed similar procedures of characterizing the multiplier as described in ref. 90. Uncertainties in U–Th isotopic data were calculated offline at the 2σ level, including corrections for blanks, multiplier dark noise, abundance sensitivity, and contents of the same nuclides in the spike solution. Corrected U–Th ages assume an initial ^230^Th/^232^Th atomic ratio of 4.4 ± 2.2 × 10^−6^, the values for a material at secular equilibrium with the bulk earth ^232^Th/^238^U value of 3.8. Most samples have high U/Th ratios and thus the corrections are negligible. A total of ∼192 U–Th dates were obtained from nine speleothem samples: D4 from Dongge Cave, SN29 from Shennong Cave, BW-1 from Kulishu Cave, Rige-3 from Rige Cave, M-1 from Mawmluh Cave, Chy-1 from Cherrapunji Caves, TON-1 from Tonnel’naya Cave, JAR-7 from Jaraguá Cave, SE09-6 from Seso Cave, and Rige-3 from Rige Cave (*SI Appendix*, Fig. S1). Dating precisions of these samples were significantly improved, and the results are listed in *SI Appendix*, Table S1.

### Annual Band Counting.

Samples Chy-1 and Rige-3 have clear annual bands observed using the confocal laser fluorescent microscopy. The annual bands were counted by the confocal laser fluorescent microscope (CLFM) (Nikon A1-plus) at the State Key Laboratory for Manufacturing Systems Engineering, Xi’an Jiaotong University, with a 40-mW, 488-nm laser line (91). Images of sample fluorescence were collected using an emission filter, which allows light with wavelengths between 500 and 550 nm (visible, green) (91). The resultant floating band-counting chronologies are consistent with U–Th dating results within uncertainties (*SI Appendix*, Fig. S3).

### Oxygen-Isotope Analysis.

A total of ∼5,080 oxygen-isotope (δ^18^O) subsamples was analyzed at Xi’an Jiaotong University, China (234 data from Chy-1, 281 data from M-1, 828 data from BW-1, 267 data from TON-1, 618 data from D4, 330 data from SN29, and 1,722 data from JAR-7), and the University of Innsbruck, Austria (800 data from SE09-6) (*SI Appendix*, Table S2). The measurements made in Innsbruck used an on-line carbonate preparation system (Gasbench II) interfaced with an isotope ratio mass spectrometer (DeltaplusXL). Analyses carried out at Xi’an Jiaotong University used a Thermo‐Finnigan MAT‐253 mass spectrometer fitted with a Kiel Carbonate Device IV. The δ^18^O values are reported in per mil (‰) deviations, relative to the VPDB standard. All subsamples were calibrated against standards, and the long‐term reproducibility for δ^18^O measurements over the course of this study (∼1 y) was typically ∼0.1‰ (1σ).

### Breakpoint Determination.

We used RAMPFIT (26) and BREAKFIT (27) algorithms to identify the onset and termination of YD objectively in various speleothem and ice-core records. The RAMPFIT algorithm measures changes in the mean of a time series by applying a “ramp” to the data using least squares and brute force. In this case, it estimates the level of a parameter for pretransition (×2) and posttransition (×1) conditions and a linear change between the change points “t1” and “t2.” Uncertainty in each estimated change point is derived from 2,000 Monte Carlo simulations using moving block bootstrap resampling. The BREAKFIT algorithm (27) employs a continuous function, consisting of two linear parts that are joined at the breakpoint. The break model is fitted to data using a weighted least-squares method with a brute-force search for the breakpoint. Statistical uncertainties in the timing of breakpoints are evaluated using 2,000 block bootstrap simulations, which preserved the distribution and serial dependence of the data over the length of a block. While both RAMPFIT and BREAKFIT provide an objective estimate for the change points in a given dataset, the choice of “fit interval” is subjective and can influence the results. The main criteria to choose analytical time intervals for both methods are as follows: 1) the interval contains two breakpoints when using RAMPFIT and one breakpoint when using BREAKFIT (26, 27), and 2) the same time intervals are used for records from the same region if possible. The selected time intervals for RAMPFIT and BREAKFIT analyses are listed in *SI Appendix*, Tables S3–S5. The analysis results and additional discussions are presented in *SI Appendix*, Fig. S4, Tables S3–S5, and *Text*. All breakpoints determined respectively via RAMPFIT and BREAKFIT algorithms for the YD agree well with visual inspections.

### Correlation Strategy.

The direct comparison between AM-AW and North Atlantic records supports the correlation strategy via matching breakpoints (36) rather than their midpoints (7, 32) (*SI Appendix*, Fig. S5).

## Supplementary Material

Supplementary File

Supplementary File

Supplementary File

## Data Availability

The data used in this study are reported in *SI Appendix*, Tables S1 and S2, or available at the National Oceanic and Atmospheric Administration (NOAA), https://www.ncdc.noaa.gov/data-access/paleoclimatology-data, and/or in previous publications.
