# Supplementary material for: Timing and structure of the Younger Dryas event and its underlying climate dynamics
Source: Proc Natl Acad Sci U S A. 2020 Sep 8;117(38):23408–17. doi: 10.1073/pnas.2007869117 (PMC7519346; doi:10.1073/pnas.2007869117)
Supplement: Supplementary File [file pnas.2007869117.sapp.pdf]

PNAS
www.pnas.org

Supplementary Information for

Timing and Structure of the Younger Dryas Event and Its Underlying Climate Dynamics

Hai Cheng[a,b,c]*, Haiwei Zhang[a], Christoph Spötl[d], Jonathan Baker[a], Ashish Sinha[e], Hanying Li[a], Miguel Bartolomé[f], Ana Moreno[g], Gayatri Kathayat[a], Jingyao Zhao[a], Xiyu Dong[a], Youwei Li[a], Youfeng Ning[a], Xue Jia[a], Baoyun Zong[a], Yassine Ait Brahim[a], Carlos Pérez-Mejías[a], Yanjun Cai[a], Valdir F. Novello[h], Francisco W. Cruz[h], Jeffrey P. Severinghaus[i], Zhisheng An[b], R. Lawrence Edwards[j,k]

[a] Institute of Global Environmental Change, Xi'an Jiaotong University, Xi'an 710054, China; [b] State Key Laboratory of Loess and Quaternary Geology, Institute of Earth Environment, Chinese Academy of Sciences, Xi'an 710061, China; [c] Key Laboratory of Karst Dynamics, MLR, Institute of Karst Geology, CAGS, Guilin, 541004, China; [d] Institute of Geology, University of Innsbruck, 6020 Innsbruck, Austria; [e] Department of Earth Sciences, California State University Dominguez Hills, CA 90747 Carson, USA; [f] Departamento de Geología. Museo Nacional de Ciencias Naturales - CSIC, Madrid, 28034, Spain; [g] Instituto Pirenaico de Ecología, Consejo Superior de Investigaciones Científicas, Spain; [h] Instituto de Geociências, Universidade de São Paulo, 05508-090 São Paulo, Brazil; [i] Scripps Institution of Oceanography, UC San Diego, CA 92093 La Jolla, USA; [j] Department of Earth and Environmental Sciences, University of Minnesota, MN 55455 Minneapolis, USA; [k] School of Geography, Nanjing Normal University, Nanjing 210023, China

*Corresponding author: Hai Cheng
Email: cheng021@xjtu.edu.cn
ORCID 0000-0002-5305-9458

This PDF file includes:

1. Supplementary Text
2. Figures S1 to S9
3. Table S1 to S5 (S1 and S2 in separated xlsx files)
4. References for SI reference citations

www.pnas.org/cgi/doi/10.1073/pnas.2007869117

1. Supplementary Text

1.1 Antarctic δ18O change around the YD termination

Oxygen-isotope ($\delta^{18}O$) data from the Greenland and Antarctic ice cores are a first-order proxy for site temperature and/or large-scale atmospheric circulation (1, 2). The Antarctic WDC $\delta^{18}O$ record exhibits two 'breakpoints' around the YD at ~12,770 and 11,900 yr BP that are broadly consistent with other high-resolution $\delta^{18}O$ records across Antarctica (Figs. S1 and S6). However, the Dome Fuji record has no breakpoint near the termination of the YD (Fig. S5).

1.2 Correlation of atmospheric CH4 with Asian Monsoon and North Atlantic records across the YD

The correlation of atmospheric CH_4 with Asian Monsoon (AM) and Greenland $\delta^{18}O$ records is illustrated in Figure S7. It is evident that centennial events during the YD, identified in the Greenland ice core, East Asian Monsoon and Indian Monsoon $\delta^{18}O$ records, are not associated with significant changes in atmospheric CH_4. A continuous CH_4 rise near the YD termination occurred from 11, 610 to 11,450 yr BP, coherent with the second step of monsoon change beyond the typical YD state, but more gradual compared to the most abrupt jump in Greenland temperature at ~11,610 yr BP, inferred from the NGRIP ice core record. These observations suggest a strong link between the AM and CH_4 (3, 4) and illustrate the inconsistency that would result from 'mid-point' matching between CH_4 and Greenland ice core $\delta^{18}O$ records.

The timing of the YD onset and termination of atmospheric CH_4 (on the WD2014 chronology, 5) is consistent with the AM records (Fig. S7) and is based on the causal linkage between CH_4 and the AM (3, 4, 6, 7), as well as the small error (~30 yr) of WDC gas-ice age difference. We thus confirm the WDC CH_4 and $\delta^{18}O$ WD2014 chronology (5) at decadal precision. This constraint is critical to resolving the lead-lag phases between northern and southern polar realms.

1.3 The possible link between Antarctic δ18O trend change at ~11,900 ±100 yr BP and the Asian Monsoon change

The WDC $\delta^{18}O$ record on the WD2014 chronology (5) shows a negative Antarctic $\delta^{18}O$ trend starting at ~11,900 ±80 yr BP. Tentatively, the East Asian and Indian Monsoon $\delta^{18}O$ records are dominated by a weak increasing trend after ~11,900 yr BP, which may be linked to this Antarctic event (8, 9) (Fig. S9).

1.4 Breakpoint determinations

The timings of the YD onset/termination in various records are defined by change point detection algorithms RAMPFIT (10) and BREAKFIT (11) and with visual inspection (see *Materials and Methods*). The anchor points marking the onset and termination of YD in the NGRIP and Seso $\delta^{18}O$ records are defined by a large abrupt drop in $\delta^{18}O$ (~2‰ within ~20 yr) at 12,870 ±30 yr BP and its termination is characterized by a two-step $\delta^{18}O$ excursion from ~11,700 ±40 to ~11,610 ±40 yr BP spanning ~90 years. These timings are consistent with the RAMPFIT and BREAKFIT algorithms based breakpoints within uncertainties (see Fig. S4 and Table S3-S5).

Therefore, we used these two anchor points in this study for global YD correlation/synchronization at sub-centennial precision.

The YD records from the Asian Monsoon and Westerlies (AM-AW) domain, tropics, and Southern Hemisphere (SH) show a large diversity in their temporal resolution and structure. The Cherrapunji $\delta^{18}O$ record provides the most robust estimate of the anchor point for the initial YD onset in AM-AW region, because of its precise age model (constrained by both annual bands and U-Th dates), high temporal resolution, and its unambiguous structure. Both RAMPFIT and BREAKFIT results provide the same breakpoint at 12,890 (±30 and ±70, respectively) yr BP (Fig. S4 and Table S3-S5), which is consistent with the NGRIP and Seso records, as well as with other records from the AM-AW domain (Fig. S4). The mean ages of breakpoints from RAMPFIT and BREAKFIT analyses marking the initial YD onset in other speleothem records are ~11,670 ±50 yr BP (Shennong), ~11,710 ±40 yr BP (Tonnel'naya), and ~11,680 ±90 yr (Kulishu). The uncertainties were calculated by combining quadratically the uncertainties from RAMPFIT and BREAKFIT results.

The YD records from the tropics are characterized by a 'V-shape' structure. Hence, the BREAKFIT algorithm is more suitable than the RAMPFIT to determine the change-points in these records. The initial YD terminations in these records, determined either by visual inspection or BREAKFIT analysis, cluster around ~12,300 yr BP (Fig. S4).

The analysis of WDC $\delta^{18}O$ record yield 'breakpoints' of 12,770 ±60 and 12,775 ±80 yr BP for the YD initial onset and 11,930 ±50 and 11,870 ±60 yr BP for the YD initial termination, from RAMPFIT and BREAKFIT algorithms, respectively. The RAMPFIT results have slightly smaller uncertainties. These results are consistent, within uncertainty, with the previous BREAKFIT analysis results (12) for the initial YD onset (12,778 ±80) and termination (11,842 ±90).

The results of breakpoint analysis for the remaining records are listed in Tables S3-S5. They are broadly consistent with the aforementioned critical breakpoints (Fig. S4), but have larger uncertainties from either age models of records or temporal errors of the breakpoints.

2. Supplementary Figures (S1 to S9)

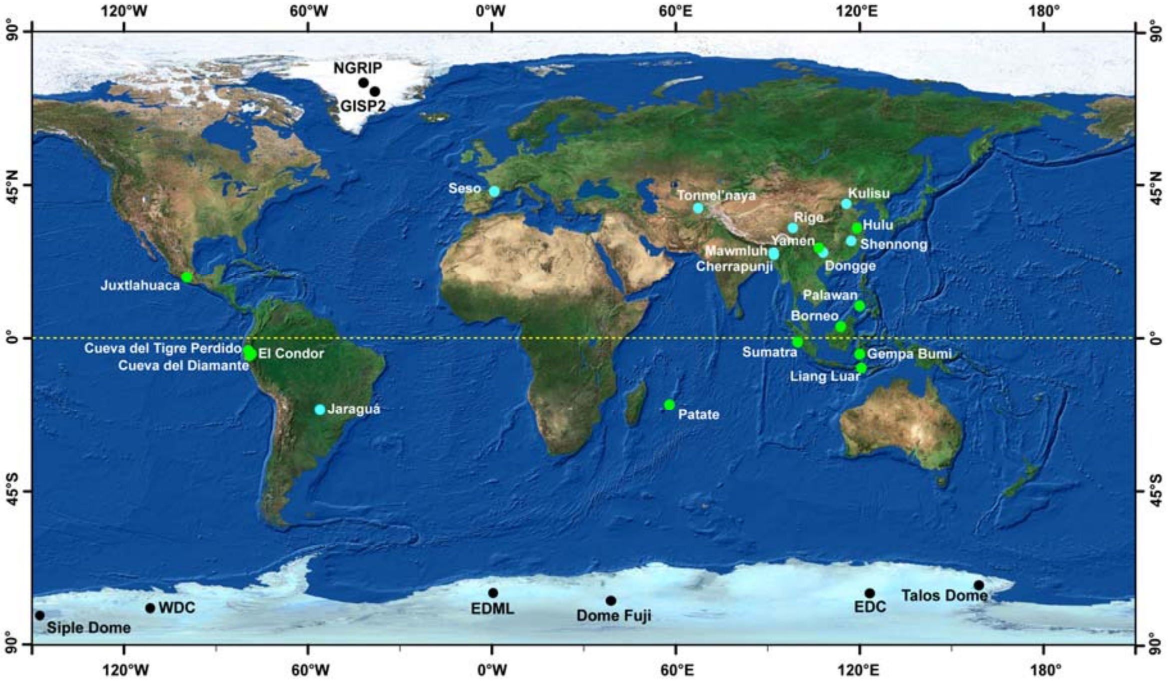

Figure S1. Proxy record locations. Blue circles show cave records presented in this study. Green circles indicate locations of other caves for comparison. Greenland and Antarctic ice core locations are shown by black dots. The map was created using ArcGIS software (ArcGIS, http://www.esrichina.com.cn/) and the world map background data are from the Environmental Systems Research Institute (ESRI).

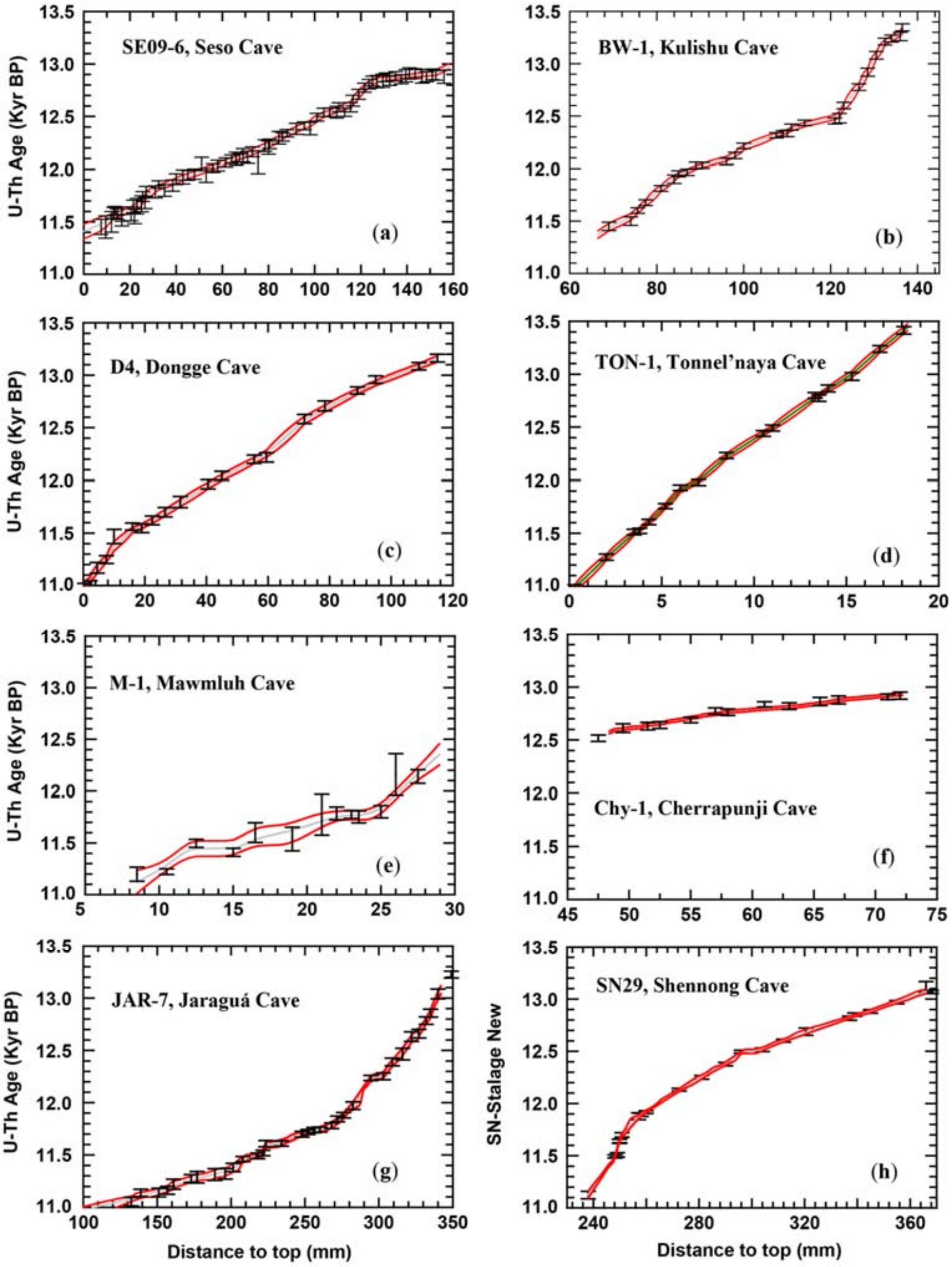

Figure S2. Age models of new speleothem records. (**A**) to (**H**) Sample SE09-6 from Seso Cave, sample BW-1 from Kulishu Cave, sample D4 from Dongge Cave, sample TON-1 from Tonnel'naya Cave, sample M-1 from Mawmluh Cave, sample Chy-1 from Cherrapunji Cave, sample JAR-7 from Jaraguá Cave, and sample SN29 from Cherrapunji Cave. Error bars show U-Th dates and errors (2σ). Age models were calculated using OxCal (13), except for M-1, JAR-7 and SN29, where StalAge (14) was used to handle reverse dates and possible hiatuses (Fig. S2). Age model for Chy-1 is obtained through the combination of annual band counting and U-Th dating methods (15) (Fig. S3).

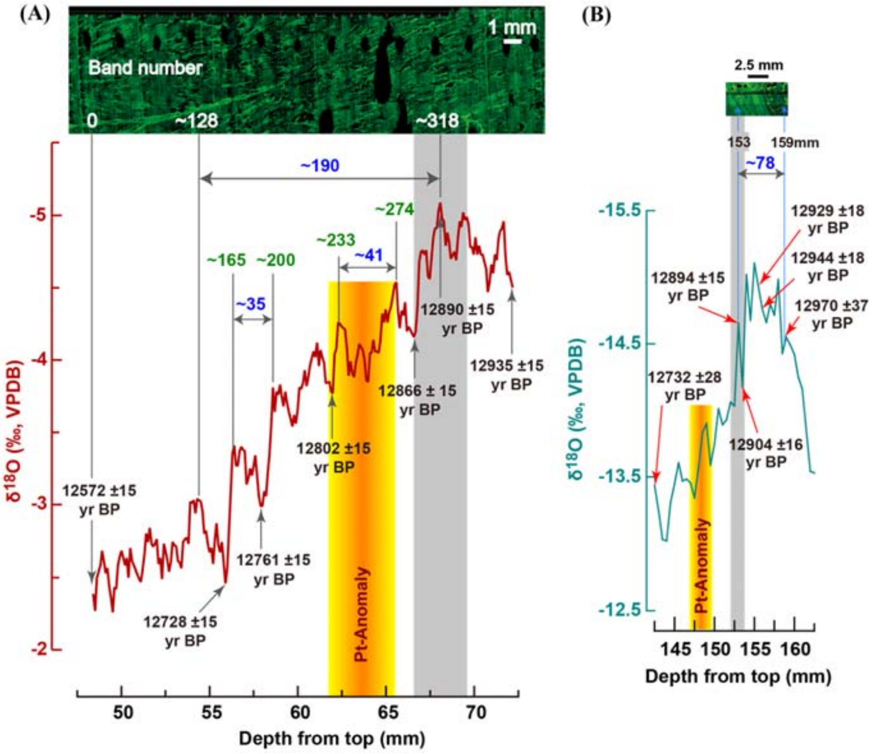

Figure S3. Age models of samples Chy-1 and Rige-3. Two images at top are laser scanning confocal microscopy photos of samples Chy-1 and Rige-3. Annual bands in the samples are clearly accountable. (**A**) Maroon curve is the plot of Chy-1 $\delta^{18}O$ versus depth. The U-Th dates (black) and band counting results (blue and green) are illustrated by labels. (**B**) Plot of Rige-3 $\delta^{18}O$ versus depth. The U-Th dates are labeled in black. The annual band counting between depth 159 and 153 mm gives an age of ~78 years (labeled as blue number) in the interval, consistent within error with U-Th dating result (~76 years). Two vertical grey bars show the initial timing of the YD onset at ~12,890 ±20 yr BP in the two records. Two vertical yellow bars indicate the timings of the Pt-anomaly at 12,820 ±20 yr BP on the GICC05 chronology (16, 17).

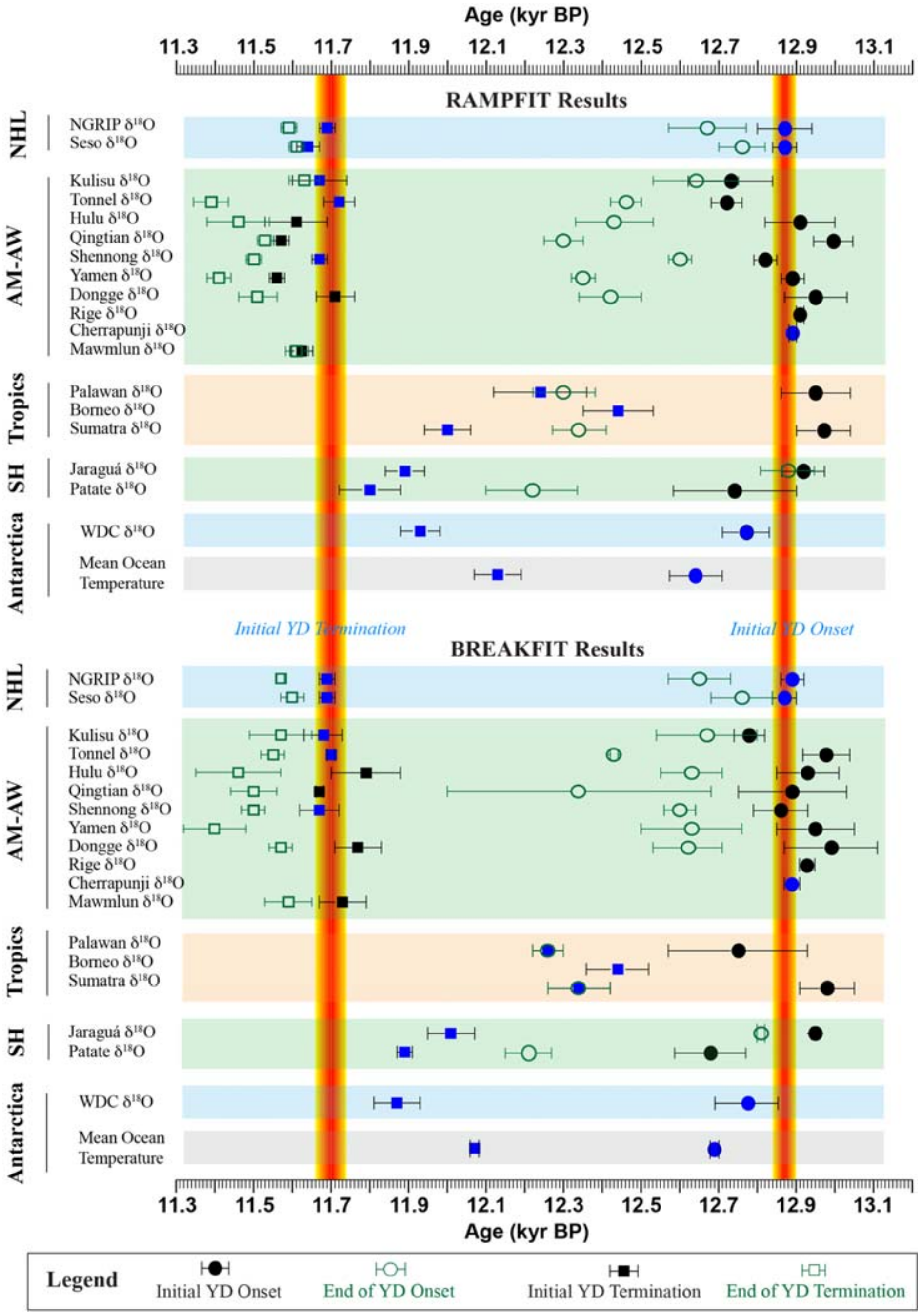

Figure S4. Breakpoint analysis results. Breakpoints (2σ error bars) were determined via RAMPFIT (upper panel) (10) and BREAKFIT (lower panel) (11) algorithms (Table S3-S5). The records analyzed were assembled into five groups: Northern high-latitude (NHL), Asian Monsoon and Westerlies (AM-AW), Tropics, Southern Hemisphere (SH), and Antarctica. The original references for the records are the same as those listed in Figures 1-4. Two vertical bars indicate initial YD onset (12,890 ±30 yr BP) and termination (11,700 ±40 yr BP), respectively. Error bars in blue depict the breakpoints used to constrain the initial YD onset and termination in the text because of their precise age model, clear structure and high temporal resolution around the time periods.

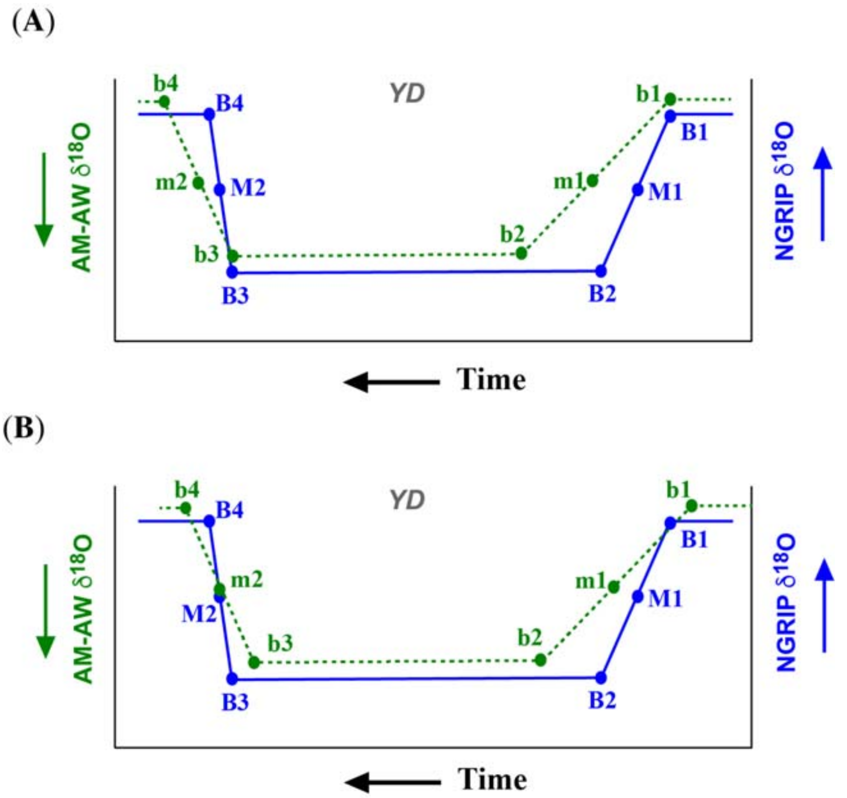

Figure S5. Schematic correlations for the YD interval. Conceptual model of AM-AW $\delta^{18}O$ (dashed green line) and NGRIP ice core $\delta^{18}O$ (solid blue line) change across the YD. B1 to B4 and b1 to b4 mark 'breakpoints' of North Atlantic and AM-AW $\delta^{18}O$ records, respectively. M1 and M2 as well as m1 and m2 depict 'mid-points' of the onset and termination in North Atlantic and AM-AW $\delta^{18}O$ records, respectively. The YD onset and termination appear more gradual in AM-AW records than in North Atlantic counterparts. (**A**) Our precisely dated records support the correlation strategy by matching 'breakpoints' b3 and B3 (18). In this case, the initial termination in AM-AW and North Atlantic records is synchronous, but North Atlantic records reach the new state (B4) earlier than AM-AW records (b4). (**B**) Conventional correlation by matching 'mid-points' m2 and M2. In this case, the initial termination in AM-AW records (b3) would lead to the corresponding North Atlantic change (B3). Additionally, this correlation would also suggest an early initial onset of the YD in the AM-AW domain (b1) relative to the North Atlantic region (B1).

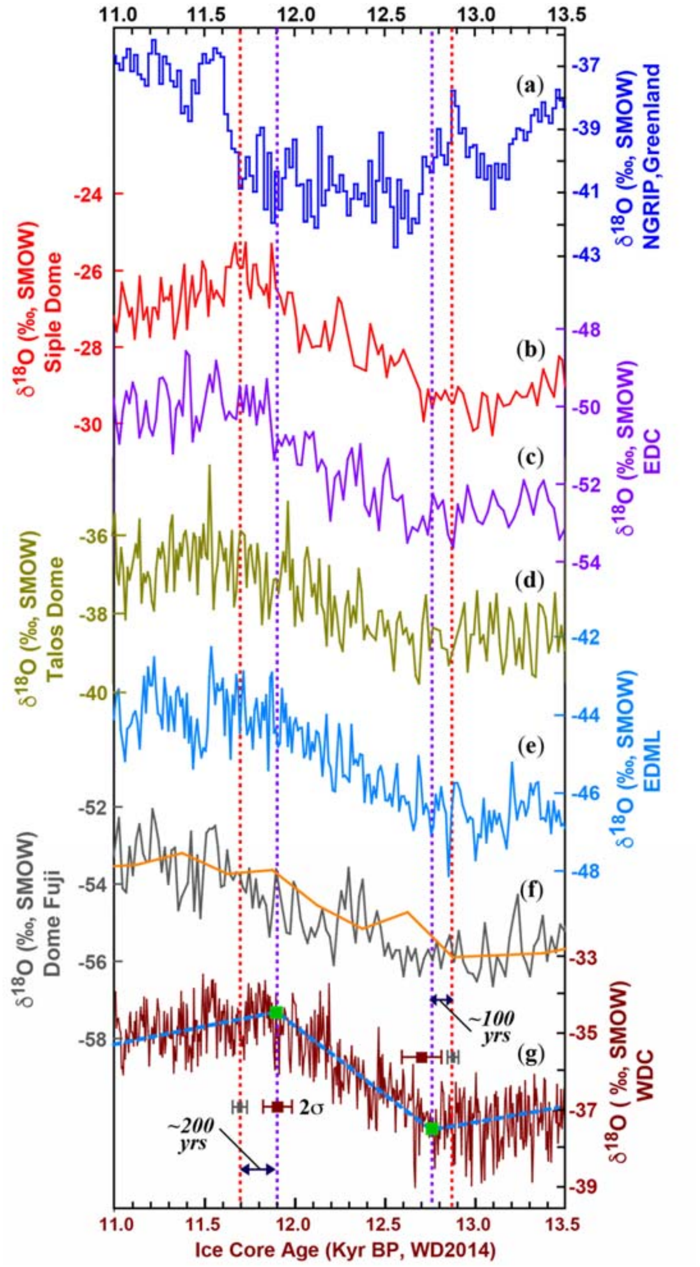

Figure S6. Antarctic temperature change across the YD. (**a**) Greenland NGRIP $\delta^{18}O$ record (17). (**b**) Antarctic ice core $\delta^{18}O$ records from Siple Dome (19). (**c**) to (**e**) Antarctic ice core $\delta^{18}O$ records from sites EDC, Talos Dome and EDML on the WD2014 chronology (7). (**f**) Antarctic ice core $\delta^{18}O$ records from Dome Fuji (orange, (19); grey, (17)). (**g**) Antarctic WDC $\delta^{18}O$ record on the WD2014 chronology (5-7). Blue lines and green squares depict trends and 'breakpoints' of the record. Two vertical red dashed lines depict the initial onset (12,870 ±30 yr BP) and initial termination (11,700 ±40 yr BP) of the YD in the NGRIP and Seso records. Two vertical purple lines indicate two breakpoints in the WDC $\delta^{18}O$ record at ~11,900 and ~12,770 yr BP. Typical 2-σ age uncertainties are depicted by error bars.

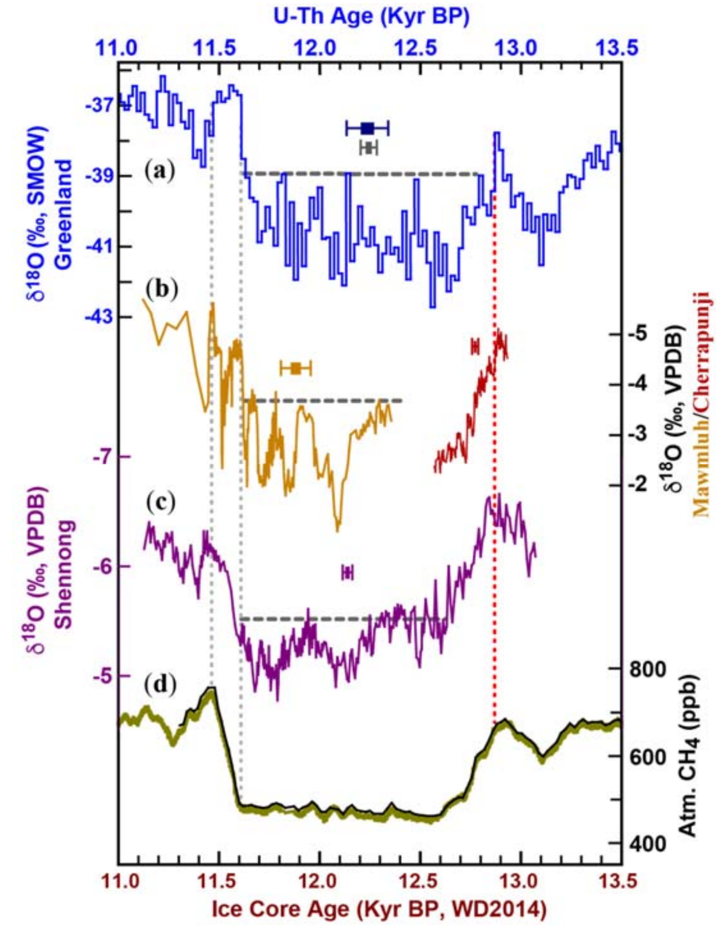

Figure S7. Methane change across the YD in the context of Greenland ice core and Asian Monsoon variations. (**a**) Greenland NGRIP $\delta^{18}O$ record (17). (**b**) Indian Monsoon $\delta^{18}O$ records (Mawmluh record in yellow and Cherrapunji record in marron). (**c**) East Asian Monsoon $\delta^{18}O$ record (Shennong record, this study). (**d**) Atmospheric methane (CH_4) records from the WDC ice core (olive, 20; black, 5). Vertical red line depicts the initial onset of the YD. Error bars depict typical age error (2σ) of each record. The blue and grey errors for the NGRIP $\delta^{18}O$ record depict NGRIP age error (GICC05 chronology, 17) and the error based on synchronization with the Seso chronology, respectively. Two vertical gray lines indicate the interval of the monsoon and CH_4 termination excursions. The horizontal dashed lines mark the peaks of centennial-scale events during the YD in Asian Monsoon and Greenland $\delta^{18}O$ records that were not associated with significant variations in atmospheric CH_4.

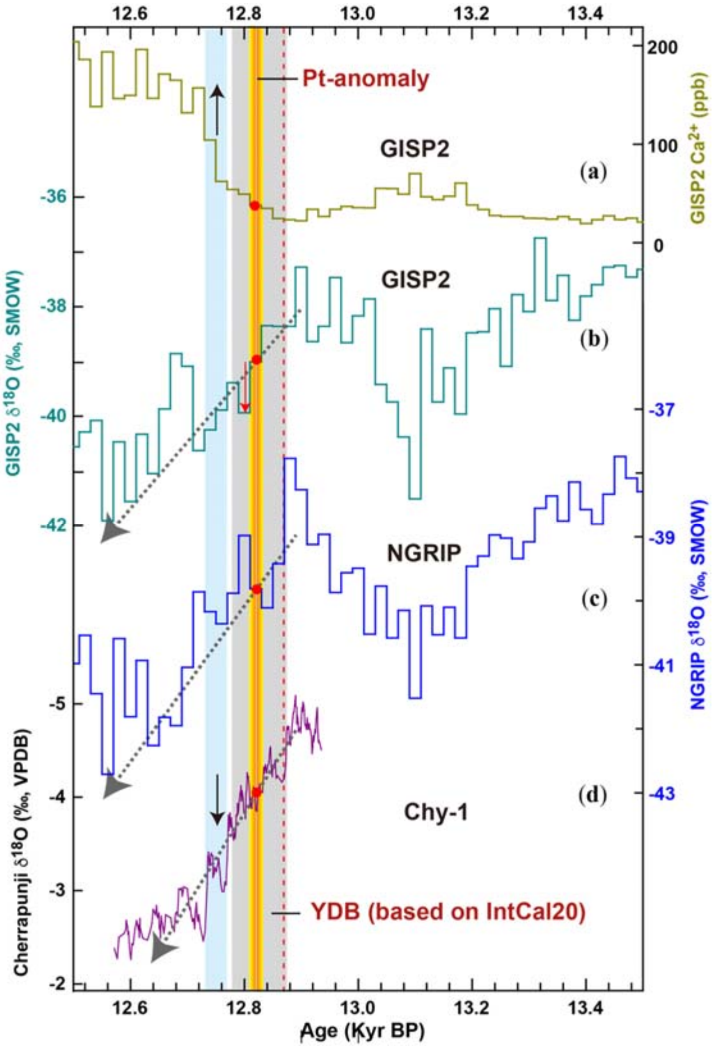

Figure S8. Temporal relation between the YDB layer and Asian Monsoon records. (**a**) and (**b**) GISP2 Ca^{2+} and $\delta^{18}O$ on GICC05 timescale (17, 21). (**c**) NGRIP $\delta^{18}O$ on GICC05 timescale (21). (**d**) Cherrapunji $\delta^{18}O$ record (this study). The vertical solid orange line depicts the Pt-anomaly in the GISP2 ice core near the initial onset of the YD (16) at ~12,820 yr BP based on synchronization with the NGRIP GICC05 chronology (17). The vertical grey bar indicates the YDB layer previously constrained to 12,835-12,735 yr BP (22, 23), and to 12,875-12,775 yr BP based on IntCal20 (24). The grey arrows depict the onset excursion of the YD. The red dashed line depicts the YD onset (abrupt decrease in NGRIP $\delta^{18}O$). The Pt-anomaly (vertical orange bar, red dots) occurred after the excursion had already carried through ~20% from the YD onset. The red arrow indicates the possible amplitude of a subsequent $\delta^{18}O$ drop in the GISP2 record plausibly caused by the extraterrestrial impact, which is minor (less than 1‰). Similarly, the YD onset excursions in GISP2 Ca^{2+} record from the same ice core, as well as the NGRIP $\delta^{18}O$ and Asian Monsoon $\delta^{18}O$ records on the GICC05 timescale, do not appear to be significantly disrupted by the Pt-anomaly event. The vertical blue bar depicts the correlation between an abrupt increase in GISP2 Ca^{2+} and an abrupt weakening in the Asian Monsoon (black arrows) (25, 26), which lags the Pt-anomaly event by ~20 years.

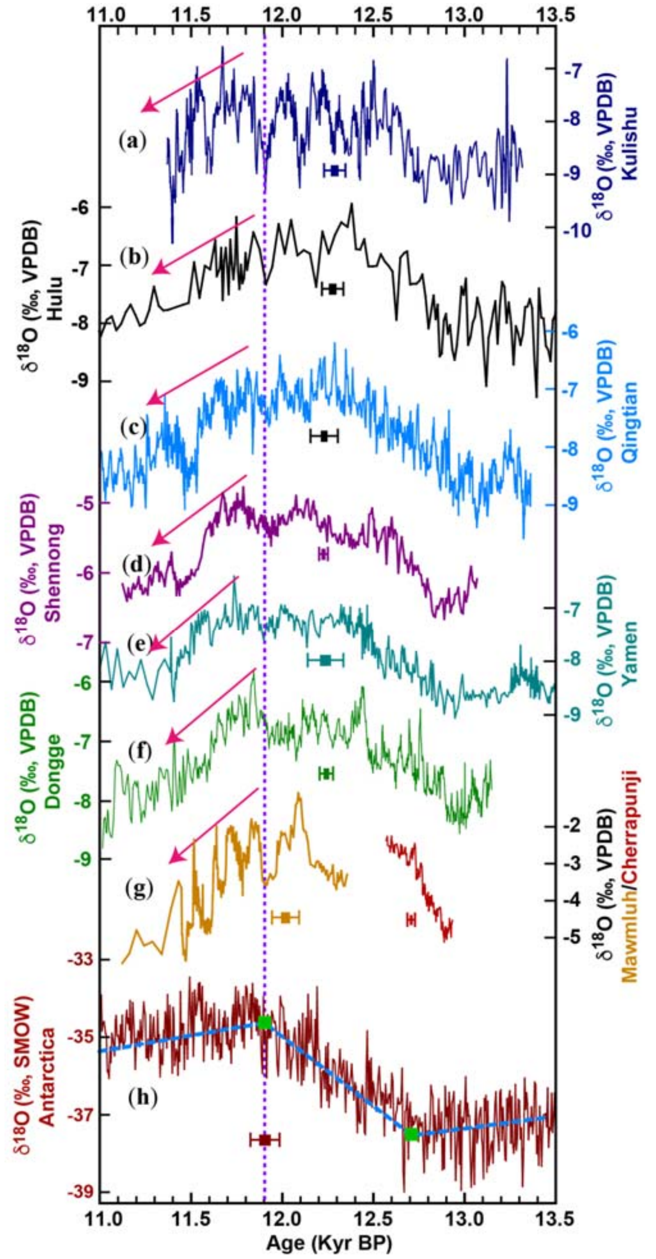

Figure S9. Comparison between Antarctic and Asian Monsoon δ^{18}O records. (**a**) to (**f**) are East Asian Monsoon δ^{18}O records from Kulishu (this study), Hulu (27), Qingtian (28), Shennong (this study), Yamen (29), and Dongge (this study) caves. (**g**) Indian Monsoon records from Mawmluh (yellow) and Cherrapunji (maroon) caves (this study). (**h**) WDC δ^{18}O record (WD2014 chronology, 5). The error bars show typical age errors of YD records (color coded). The dashed vertical purple line depicts the initial onset (~11,900 ±80 yr BP) of the Antarctic cooling. The arrows show the weak monsoon trend of the Asian Monsoon after ~11,900 yr BP.

3. Supplemental Tables S1 to S5 (S1 and S2 in separated xlsx files)

Table. S3 Results of "RAMPFIT"

Stages	Regions	Analytical interval (yr BP)	Proxies	t_1 (yr BP)*	Error (yr)	t_2 (yr BP)*	Error (yr)
YD Onset	Northern high latitude	12200-13000	NGRIP $\delta^{18}O$	12870	70	12670	100
			Seso $\delta^{18}O$	12870	30	12760	60
	AM-AW	12200-13100	Hulu $\delta^{18}O$	12910	90	12430	100
			Kulishu $\delta^{18}O$	12730	110	12640	110
			Yamen $\delta^{18}O$	12890	30	12350	30
			Dongge $\delta^{18}O$	12950	80	12420	80
			Cherrapunji $\delta^{18}O$	12890	10	—	—
			Tonnel $\delta^{18}O$	12720	40	12460	40
			Shennong $\delta^{18}O$	12820	30	12600	30
		12100-13000	Qingtian $\delta^{18}O$	12995	50	12300	50
		12850-12950	Rige $\delta^{18}O$	12910	10	—	—
	Tropical region	12200-13100	Palawan $\delta^{18}O$	12950	90	12300	80
			Sumatra $\delta^{18}O$	12970	70	12340	70
	Southern Hemisphere	12400-13100	Jaraguá $\delta^{18}O$	12920	55	12880	70
		12100-13000	Patate $\delta^{18}O$	12740	160	12220	150
	Global signal	12200-13100	WDC CH_4	12870	20	12630	20
YD Termination	Northern high latitude	11500-11900	NGRIP $\delta^{18}O$	11690	20	11590	20
			Seso $\delta^{18}O$	11640	30	11610	20
	AM-AW	11300-12000	Hulu $\delta^{18}O$	11610	80	11460	80
			Dongge $\delta^{18}O$	11710	50	11510	50
			Yamen $\delta^{18}O$	11560	20	11410	30
			Qingtian $\delta^{18}O$	11570	20	11530	20
			Mawmlun $\delta^{18}O$	11623	30	11611	30
		11300-11700	Shennong $\delta^{18}O$	11670	20	11500	20
		11530-11850	Kulishu $\delta^{18}O$	11670	70	11630	40
		11300-11900	Tonnel $\delta^{18}O$	11720	40	11390	45
	Tropical region	11600-12600	Borneo $\delta^{18}O$	12440	90	—	—
		11800-12500	Palawan $\delta^{18}O$	12240	120	—	—
		11600-12400	Sumatra $\delta^{18}O$	12000	60	—	—
	Southern Hemisphere	11700-11900	Jaraguá $\delta^{18}O$	11890	50	—	—
		11600-12000	Patate $\delta^{18}O$	11800	80	—	—
	Global signal	11400-11900	WDC CH_4	11610	10	11480	10
	Antarctica	11300-13100	WDC $\delta^{18}O$	12770	60	11930	50
	Ocean	11300-13100	Mean Ocean Temperature	12640	70	12130	60

*t_1: timing for initial onset/termination; t_2: timing for end of onset/termination
For WDC $\delta^{18}O$ record and Mean Ocean Temperature, t_1 indicates the timing of initial transition, t_2 indicates the timing of finished transition.
For Tables S3-5, The termination of records in Tropical region and subtropical region of southern hemisphere quoted in this study undergone many stages, and we only show the timings of the initial termination.
Errors are 2σ from RAMPFIT and BREAKFIT. Errors that are less than 10 years are regarded as 10 years.

Table. S4 Results of "BREAKFIT" analyses for YD onset

Stages	Regions	Analytical interval (yr BP)	Proxies	T (yr BP)	Error (yr)
Beginning of YD Onset	Northern high latitude	12700-13000	NGRIP $\delta^{18}O$	12890	30
			Seso $\delta^{18}O$	12870	30
	AM-AW	12500-13100	Hulu $\delta^{18}O$	12930	80
			Yamen $\delta^{18}O$	12950	100
			Dongge $\delta^{18}O$	12990	120
		12600-13000	Kulishu $\delta^{18}O$	12780	40
		12700-end	Cherrapunji $\delta^{18}O$	12890	20
		12700-13100	Tonnel $\delta^{18}O$	12980	60
		12500-13000	Shennong $\delta^{18}O$	12860	70
			Qingtian $\delta^{18}O$	12890	140
		12850-12950	Rige $\delta^{18}O$	12930	20
	Tropical region	12700-13200	Palawan $\delta^{18}O$	12750	180
			Sumatra $\delta^{18}O$	12980	70
	Southern Hemisphere	12800-13000	Jaraguá $\delta^{18}O$	12950	10
		12500-12900	Patate $\delta^{18}O$	12680	90
	Global signal	12700-13000	WDC CH_4	12900	10
End of YD Onset	Northern high latitude	12600-12900	NGRIP $\delta^{18}O$	12650	80
			Seso $\delta^{18}O$	12760	80
	AM-AW	12300-12700	Hulu $\delta^{18}O$	12630	80
			Kulishu $\delta^{18}O$	12670	130
			Yamen $\delta^{18}O$	12630	130
			Dongge $\delta^{18}O$	12620	90
			Tonnel $\delta^{18}O$	12430	10
			Shennong $\delta^{18}O$	12600	40
		12100-12700	Qingtian $\delta^{18}O$	12340	340
	Tropical region	12100-12600	Palawan $\delta^{18}O$	12260	40
			Sumatra $\delta^{18}O$	12340	80
	Southern Hemisphere	12700-12900	Jaraguá $\delta^{18}O$	12810	10
		12100-12400	Patate $\delta^{18}O$	12210	60
	Global signal	12300-12800	WDC CH_4	12610	20
	Antarctica	12200-13300	WDC $\delta^{18}O$	12775	80
	Ocean	12200-13300	Mean Ocean Temperature	12690	10

Table. S5 Results of "BREAKFIT" analyses for YD termination

Stages	Regions	Analytical interval (yr BP)	Proxies	t (yr BP)	Error (yr)
Beginning of YD Termination	Northern high latitude	11600-11800	NGRIP $\delta^{18}O$	11690	20
			Seso $\delta^{18}O$	11690	20
	AM-AW	11600-12000	Hulu $\delta^{18}O$	11790	90
		11500-11800	Dongge $\delta^{18}O$	11770	60
			Yamen $\delta^{18}O$	11750	90
			Qingtian $\delta^{18}O$	11670	10
			Mawmlun $\delta^{18}O$	11730	60
		11600-11800	Shennong $\delta^{18}O$	11670	50
			Kulishu $\delta^{18}O$	11680	50
			Tonnel $\delta^{18}O$	11700	10
	Tropical region	12100-12600	Borneo $\delta^{18}O$	12440	80
			Palawan $\delta^{18}O$	12260	40
			Sumatra $\delta^{18}O$	12340	80
	Southern Hemisphere	11700-12100	Jaraguá $\delta^{18}O$	12010	60
			Patate $\delta^{18}O$	11890	20
	Global signal	11500-11800	WDC CH_4	11610	10
End of YD Termination	Northern high latitude	11500-11700	NGRIP $\delta^{18}O$	11570	10
			Seso $\delta^{18}O$	11600	30
	AM-AW	11200-11700	Hulu $\delta^{18}O$	11460	110
			Dongge $\delta^{18}O$	11570	30
			Yamen $\delta^{18}O$	11400	80
			Qingtian $\delta^{18}O$	11500	60
			Mawmlun $\delta^{18}O$	11590	60
			Shennong $\delta^{18}O$	11500	30
			Kulishu $\delta^{18}O$	11570	80
			Tonnel $\delta^{18}O$	11550	30
	Global signal	11300-11600	WDC CH_4	11470	10
	Antarctica	10700-12700	WDC $\delta^{18}O$	11870	60
	Ocean	10700-12700	Mean Ocean Temperature	12070	10

4. References

1. J. Jouzel, R. B. Alley, K. M. Cuffey, W. Dansgaard, P. Grootes, G. Hoffmann, S. J. Johnsen, R. D. Koster, D. Peel, C. A. Shuman, M. Stievenard, M. Stuiver, J. White, Validity of the temperature reconstruction from water isotopes in ice cores. *J. Geophys. Res*. **102**, 26471–26487 (1997).

2. B. R. Markle, E. J. Steig, C. Buizert, S. W. Schoenemann, C. M. Bitz, T. J. Fudge, J. B. Pedro, Q. Ding, T. R. Jones, J. W. C. White. Global atmospheric teleconnections during Dansgaard- Oeschger events. *Nat. Geosci*. **10**, 36–40 (2017).

3. H. Cheng, R. L. Edwards, Y. J. Wang, X. G. Kong, Y. F. Ming, C. D. Gallup, M. J. Kelly, X. F. Wang, and W. G. Liu, A penultimate glacial monsoon record from Hulu Cave and two-phase glacial terminations. *Geology* **34**, 217–220 (2006).

4. H. Cheng, R. L. Edwards, W. S. Broecker, G. H. Denton, X. G. Kong, Y. J. Wang, R. Zhang, X. F. Wang, Ice Age Terminations. *Science* **326**, 248–252 (2009).

5. M. Sigl, T. J. Fudge, M. Winstrup, J. Cole-Dai, D. Ferris, J. R. McConnell, K. C. Taylor, K. C. Welten, T. E. Woodruff, F. Adolphi, M. Bisiaux, E. J. Brook, C. Buizert, M. W. Caffee, N. W. Dunbar, R. Edwards, L. Geng, N. Iverson, B. Koffman, L. Layman, O. J. Maselli, K. McGwire, R. Muscheler, K. Nishiizumi, D. R. Pasteris, R. H. Rhodes, T. A. Sowers, The WAIS Divide deep ice core WD2014 chronology - Part 2: Annual-layer counting (0-31 ka BP). *Clim. Past* **12**, 769–786 (2016).

6. C. Buizert, K. M. Cuffey, J. P. Severinghaus, D. Baggenstos, T. J. Fudge, E. J. Steig, B. R. Markle, M. Winstrup, R. H. Rhodes, E. J. Brook, T. A. Sowers, G. D. Clow, H. Cheng, R. L. Edwards, M. Sigl, J. R. McConnell, K. C. Taylor, The WAIS-Divide deep ice core WD2014 chronology: 2. Methane synchronization (68-31 ka BP) and the gas age-ice age difference. *Clim. Past* **11**, 153–173 (2015).

7. C. Buizert, M. Sigl, M. Severi, B. R. Markle, J. J. Wettstein, J. R. McConnell, J. B. Pedro, H. Sodemann, K. Goto-Azuma, K. Kawamura, Abrupt ice-age shifts in southern westerly winds and Antarctic climate forced from the north. *Nature* **63**, 681–685 (2018).

8. Y. Cai, Z. An, H. Cheng, L. R. Edwards, M. J. Kelly, W. Liu, X. Wang, C. C. Shen, High-resolution absolute-dated Indian Monsoon record between 53 and 36 ka from Xiaobailong Cave, southwestern China. *Geology* **34**, 621–624 (2006).

9. E. J. Rohling, Q. S. Liu, A. P. Roberts, J. D. Stanford, S. O. Rasmussen, P. L. Langen, M. Siddall, Controls on the East Asian monsoon during the last glacial cycle, based on comparison between Hulu Cave and polar ice-core records. *Quat. Sci. Rev.* **28**, 3291–3302 (2009).

10. M. Mudelsee, Ramp function regression: a tool for quantifying climate transitions. *Comput. Geosci*. **26**, 293–307 (2000).

11. M. Mudelsee, Break function regression: a tool for quantifying trend changes in climate time series. *Eur. Phys. J. Spec. Topics* **174**, 49–63 (2009).

12. WAIS Divide Project Members. Precise interpolar phasing of abrupt climate change during the last ice age. *Nature* **520**, 661–665 (2015).

13. Ramsey, C., Lee, S. Recent and Planned Developments of the Program OxCal. *Radiocarbon* **55**, 2–3 (2013).

14. Scholz, D., Hoffmann, D.L. StalAge–An algorithm designed for construction of speleothem age models. *Quat. Geochronol.* **6**, 369–382 (2011).

15. Domínguez-Villar, D, Baker, A., Fairchild, I. J., Edwards, R. L. A method to anchor floating chronologies in annually laminated speleothems with U–Th dates. *Quat. Geochronol.* **14**, 57-66 (2012).

16. M. Petaev, S. Huang, S. B Jacobsen, A. Zindler, Large Pt anomaly in the Greenland ice core points to a cataclysm at the onset of Younger Dryas. *Proc. Natl. Acad. Sci. USA* **110**, 12917–12920 (2013).

17. S.O. Rasmussen, M. Bigler, S.P. Blockley, T. Blunier, S.L. Buchardt, H.B. Clausen, I. Cvijanovic, D. Dahl-Jensen, S. J. Johnsen, H. Fischer, V. Gkinis, M. Guillevic, W.Z. Hoek, J. J. Lowe, J. B. Pedro, T. Popp, I. K. Seierstad, J. P. Steffensen, A. M. Svensson, P. Vallelonga, B. M. Vinther, M. J. C. Walker, J. J. Wheatley, M. Winstrup, A stratigraphic framework for abrupt climatic changes during the Last Glacial period based on

three synchronized Greenland ice-core records: re- fining and extending the INTIMATE event stratigraphy. *Quat. Sci. Rev.* **106**, 14–28 (2014).

18. W. Du, H. Cheng, Y. Xu, X. Yang, P. Zhang, L. Sha, H. Li, X. Zhu, M. Zhang, N. M. Stríkis, F. W. Cruz, R. L. Edwards, H. Zhang, Y. Ning, Timing and structure of the weak Asian Monsoon event about 73000 years ago. *Quat. Geochronol.* **53**, 101003 (2019).
19. WAIS Divide Project Members. Onset of Deglacial Warming in West Antarctica Driven by Local Orbital Forcing, *Nature* **500**, 440–444 (2013).
20. R. H. Rhodes, E. J. Brook, J. C. H. Chiang, T. Blunier, O. J. Maselli, J. R. McConnell, D. Romanini, J. P. Severinghaus, Enhanced tropical methane production in response to iceberg discharge in the North Atlantic. *Science* **348**, 1016–1019 (2015).
21. S. O. Rasmussen, K. K. Andersen, A. M. Svensson, J. P. Steffensen, B. M. Vinther, H. B. Clausen, M. L. Siggaard-Andersen, S. J. Johnsen, L. B. Larsen, D. Dahl-Jensen, M. Bigler, R. R. Thlisberger, H. Fischer, K. Goto-Azuma, M. E. Hansson, U. Ruth, A new Greenland ice core chronology for the last glacial termination. *J. Geophys. Res.* **111** (D6), 907–923 (2006).
22. J. P. Kennett, D. J. Kennett, B. J. Culleton, J. E. A. Tortosa, J. L. Bischoff, T E. Bunch, I. R. Daniel, J. M. Erlandson, D. Ferraro, R. B. Firestone. Bayesian chronological analyses consistent with synchronous age of 12,835-12,735 Cal B.P. for Younger Dryas boundary on four continents. *Proc. Nat. Acad. Sci. USA* **112**, E4344–4353 (2015).
23. P. Reimer, P. J. Reimer, E. Bard, A. Bayliss, J. W. Beck, P. G. Blackwell, C. B. Ramsey, C. E. Buck, H. Cheng, R. L. Edwards, M. Friedrich, P. M. Grootes, T. P. Guilderson, H. Haflidason, I. Hajdas, C. Hatté, T. J. Heaton, D. L. Hoffmann, A. G. Hogg, K. A. Hughen, K. F. Kaiser, B. Kromer, S. W. Manning, M. Niu, R. W. Reimer, D. A. Richards, E. M. Scott, J. R. Southon, R. A. Staff, C. S. M. Turney, Jvd. Plicht, IntCal13 and Marine13 radiocarbon age calibration curves 0-50,000 years cal BP. *Radiocarbon* **55**, 1869–87 (2013).
24. P. Reimer, W. E. N. Austin, E. Bard, A. Bayliss, P. G. Blackwell, C. B. Reimer, M. Butzin, H. Cheng, R. L. Edwards, M. Friedrich, P. M. Grootes, T. P. Guilderson, I. Hajdas, T. J. Heaton, A. G. Hogg, K. A. Hughen, B. Kromer, S. W. Manning, R. Muscheler, J. G. Palmer, M., C. Pearson, H. van der Plicht, R. W. Reimer, D. A. Richards, E. M. Scott, J. R. Southon, C. S. M. Turney, L. Wacher, The IntCal20 Northern Hemisphere radiocarbon age calibration curve (0-55 cal kBP). *Radiocarbon* In press.
25. J. C. H. Chiang, I. Y. Fung, C. -H. Wu, Y. J. Cai, J. P. Edman, Y.W. Liu, J. A. Day, T. Bhattacharya, Y. Mondal, C.A. Labrousse, Role of seasonal transitions and westerly jets in East Asian paleoclimate. *Quat. Sci. Rev.* **108,** 111–129 (2015).
26. H. Cheng, C. Spötl, S. F. Breitenbach, A. Sinha, J. A. Wassenburg, K.P. Jochum, D. Scholz, X. Li, L. Yi, Y. Peng, Y. Lv, P. Zhang, A. Votintseva, V. Loginov, Y. Ning, G. Kathayat, R. L. Edwards, Climate variations of Central Asia on orbital to millennial timescales. *Sci. Rep.* **6**, 36975 (2016).
27. H. Cheng, R. L. Edwards, J. Southon, K. Matsumoto, J. M. Feinberg, A. Sinha, W. Zhou, H. Li, X. Li, Y. Xu, S. Chen, M. Tan, Q. Wang, Y. Wang, Y. Ning, Atmospheric 14C/12C changes during the last glacial period from Hulu Cave. Science 362, 1293–1297 (2018).
28. D. Liu, Y. Wang, H. Cheng, X. Kong, S. Chen, Centennial–scale Asian monsoon variability during the mid–Younger Dryas from Qingtian cave, central China. *Quat. Res.* **80**, 199–206 (2013).
29. Y. Yang, D. Yuan, H. Cheng, M. Zhang, J. Qin, Y. Lin, X. Zhu, R. L. Edwards, Precise dating of abrupt shifts in the Asian Monsoon during the last deglaciation based on stalagmite data from Yamen Cave, Guizhou Province, China. *Sci. China-Earth Sci.* **53**, 633–641 (2010).
